# Author Correction: India’s agroecology programme, ‘Zero Budget Natural Farming’, delivers biodiversity and economic benefits without lowering yields

**DOI:** 10.1038/s41559-025-02931-0

**Published:** 2025-11-19

**Authors:** Iris Berger, Ajit Kamble, Oscar Morton, Varsha Raj, Sayuj R. Nair, David P. Edwards, Hannah S. Wauchope, Viral Joshi, Parthiba Basu, Barbara Smith, Lynn V. Dicks

**Affiliations:** 1 Department of Zoology, University of Cambridge, Cambridge, UK; 2 Conservation Research Institute, University of Cambridge, Cambridge, UK; 3 Independent Researcher, Mumbai, India; 4 Department of Plant Sciences and Centre for Global Wood Security, University of Cambridge, Cambridge, UK; 5 School of Biosciences, University of Sheffield, Sheffield, UK; 6 Global Change Institute, School of Geosciences, University of Edinburgh, Edinburgh, UK; 7 Indian Institute of Science Education and Research Tirupati, Tirupati, Andhra Pradesh India; 8 Centre for Agroecology and Pollination Studies, Department of Zoology, University of Calcutta, Kolkata, West Bengal India; 9 Centre for Agroecology, Water and Resilience, Coventry University, Coventry, UK

**Keywords:** Agroecology, Tropical ecology, Environmental economics, Sustainability

Correction to: *Nature Ecology & Evolution* 10.1038/s41559-025-02849-7, published online 18 September 2025.

In the version of this article initially published, in Eqs. (1)–(3), the term “offset (log(EA))” appeared originally as “offset (EA),”; in Eqs. (1)–(4), the equals signs following log and logit terms appeared originally as approximately, “~”; in Eq. (4), “*f* (*y*_*af*_) ~” appeared originally as “*f* (*y*_*af*_) =.” The equations are amended in the HTML and PDF versions of the article.

