# Supplementary material for: India’s agroecology programme, ‘Zero Budget Natural Farming’, delivers biodiversity and economic benefits without lowering yields
Source: Nat Ecol Evol. 2025 Sep 19;9(11):2057–68. doi: 10.1038/s41559-025-02849-7 (PMC12592218; doi:10.1038/s41559-025-02849-7)
Supplement: Supplementary file 1 — Supplementary Notes, Figures and Tables. [file 41559_2025_2849_MOESM1_ESM.pdf]

nature ecology & evolution

Supplementary information https://doi.org/10.1038/s41559-025-02849-7

India's agroecology programme, 'Zero Budget Natural Farming', delivers biodiversity and economic benefits without lowering yields

In the format provided by the authors and unedited

Table of contents

Supplementary Notes.....2
1. Study context: Zero Budget Natural Farming.....2
2. Site selection in the field.....4
3. Farmer interviews, yield and profit calculations.....8
4. Bird surveys.....12
5. Statistical matching.....14
6. ZBNF impact on yield: robustness checks.....20
7. Trade-offs between productivity and bird conservation: additional notes.....21
8. Bray-Curtis dissimilarity index at the square-level.....22
References.....23
Supplementary Figures.....28
Supplementary Tables.....40

Supplementary Notes

1. Study context: Zero Budget Natural Farming

Description of Zero Budget Natural Farming (ZBNF)

ZBNF, also sometimes referred to as Andhra Pradesh Community Managed Natural Farming (APCNF) in the southeast Indian state of Andhra Pradesh, is an agroecological farming system that aims to improve farm viability by boosting yields and reducing costs through the use of non-synthetic inputs sourced locally[1]. 'Zero budget' refers to the intention of drastically reducing the need for financial inputs and thus ending the farmers' dependence on external synthetic inputs and agricultural credit[1]. 'Natural farming' refers to the agroecological basis on which these expenditure cuts are to be achieved. ZBNF has the potential to considerably improve soil health and consequently the efficiency of nutrient and water use, implying a greater efficiency of crop production[2]. In addition to the 'four wheels', farmers are encouraged to plant live fences, keep cover crops, and design fields using a 'five layer' multi-cropping model which involves diversifying crop plants and integrating trees[3].

Self-reports on the direction of change in yield and income after switching to ZBNF have been documented to be predominantly positive[4]. Field samples taken suggest that ZBNF farmers have higher yields and net incomes than non-ZBNF farmers[1,5]. Controlled field plot experiments similarly suggest yield benefits, albeit the magnitude of the effect is crop- and region-dependent[6]. Duddigan et al[6] propose that ZBNF yield benefits are mediated by mulching's positive effect on earthworm populations and soil moisture, and leading to reduced soil temperature. Whilst ZBNF and conventional farming did not differ in their content of most macronutrients[6],

others have warned that long-term yield penalties may occur when switching from high-input systems to ZBNF and stressed that a greater understanding of the potential mechanisms of nitrogen (and other macro- and micronutrient) supply to crops in ZBNF systems is needed[2].

ZBNF is considered by many government officials the future for sustainable farming in India[7], and it now represents one of the largest ‘experiments’ of agroecological intensification globally[2]. It has also been identified as being highly relevant to global biodiversity conservation[8]. However, the strong governmental support is not underpinned by a detailed understanding of how ZBNF affects above- and below-ground ecosystem functions and services, and biodiversity.

ZBNF as an agricultural system redesign policy in Andhra Pradesh

The yields of conventional high-input farming systems in India have been declining since the 1990s[9,10]. Andhra Pradesh (AP), a state in the south-east, has had some of the highest per-capita application rates of synthetic pesticides in India[1] as well as the highest rate of indebtedness among farmers[11]. In response to this agrarian crisis, the government of AP is aiming to roll out ZBNF to all six million farmers in the state by 2030[12]. The AP Department of Agriculture set up a dedicated non-profit *Rythu Sadhikara Samstha* (RySS – Farmer’s Empowerment Organisation) in 2016 to facilitate the state-led transition towards ZBNF through extension and training programmes[1].

RySS is present in all districts and subdistricts (‘*mandals*’) in AP. They aim to target farmers from varied socio-economic settings, from high agrichemical and financial input farmers to subsistence tribal farmers, albeit the strength of exposure in each area is influenced, for example, by civil society networks and local leaders[11].

Moreover, as ZBNF adoption is voluntary, the early adopters of ZBNF may differ from the average farmer in AP with regards to characteristics that influence ZBNF programme participation and the yield, profit, and/or biodiversity outcomes. Thus, important social and economic attributes could vary between ZBNF and agrichemical farmers and their landholdings, and these must be addressed if robust inferences are to be drawn.

2. Site selection in the field

To identify the most suitable districts in Andhra Pradesh (AP) within which to conduct our study, we used forest cover (Copernicus Global Land Cover Layer, CGLS-LC100[13]) and Protected Area spatial data to select districts with large, forested areas, with at least some of them not strictly protected (IUCN category II, as obtaining permits to work in strictly protected forest sites would have not been possible and because they are unlikely to represent the management conditions experienced by the farmland sites prior to their conversion to agriculture). The following districts in northern AP were selected: East Godavari, West Godavari, Vizianagaram, Visakhapatnam, and Srikakulam. This region, totalling around 44,000 km^2, is sufficiently large and socio-ecologically varied to ensure that enough sampling sites fulfilling certain criteria (see below) could be identified and that we could estimate ZBNF programme impact across conditions, but small enough to make travelling between sites feasible and to reduce bird species turnover and the possibility for unobserved confounders. The agrichemical farming practices that the ZBNF programme aims to replace are varied, as are the socio-economic contexts and biophysical settings. Thus, in order to evaluate the impacts of the ZBNF programme quantitatively we had to sample ZBNF and non-ZBNF sites that are representative of all feasible regional agricultural systems and landscape structures.

ZBNF sites ('squares')

For these districts, RySS provided us with a list of 206 'ZBNF villages', which are villages where most farmers (approximately >80%) are practicing ZBNF. We removed villages where the farmers had been practicing ZBNF for less than four years (i.e., adoption occurred after 2017), which left us with 101 villages. We contacted the relevant RySS sub-district managers to enquire whether large continuous areas (minimum 500m x 500m; 0.25 km^2) exclusively farmed using ZBNF practices could be found within the boundaries of the given ZBNF village. Since farmers would frequently trial ZBNF on only a proportion of their land before converting completely, only 39 villages met this requirement.

We chose 25 ha (0.25 km^2) as we believed that for many bird species this would be the minimum scale at which the farming practice impact on density would manifest itself (see Hill and Hamer, 2004; Kirk et al., 2020). Whilst a larger area would have been desirable, it was impossible to find enough sites where ZBNF was practiced exclusively. In recognition that 25ha is a relatively small area, we also required ZBNF to be the dominant farming practice in the surrounding landscape; specifically, at least 75% of the farmed area in the 5 km radius surrounding the centre of square. Despite the squares relatively small sizes we regard it as appropriate to refer to them as 'landscapes' given their structural heterogeneity and socio-ecological complexity. In an agroecosystem context, 'landscape-scale' tends not to be defined in absolute geographical scales but is the scale at which interrelated environmental, economic, and social processes operate, making it highly context-dependent[14]. In our smallholder-dominated system, 'landscape-scale' thus encompasses relatively few hectares.

We mapped the 39 ZBNF villages and overlaid the Copernicus Global Land Cover Layer (CGLS-LC100[13]) with digital elevation data (SRTM V3[15]), slope data (SRTM V3[15]), annual rainfall data[16], mean annual temperature data[17], and the Harmonized World Soil Database[18] in Google Earth Engine (GEE). We removed villages that are situated less than 3 km from the nearest land use of a different type (forest, urban, etc.) to minimise edge effects, and sites over 600m in elevation as no agrichemical farming occurs at these altitudes. This left us with 28 villages. None of the ZBNF villages had slope, rainfall, temperature, or soil type characteristics that were unrepresentative of the region, implying that agrichemical and forest sites with similar attributes should be identifiable.

Using Google Earth imagery, we examined the amount of natural and semi-natural habitat patches within a 1 km radius of the village. We conducted visits to the 28 ZBNF villages. We randomised the order of our visits, whilst ensuring that we captured the full spectrum of embedded native vegetation cover and that we equally sampled from ‘plain’ and ‘tribal’ areas. ‘Tribal’ areas are characterised by traditional and indigenous farming practices, low accessibility, and a high proportion of subsistence farmers. In contrast, ‘plain’ areas, which are nearer to the coast and better connected to agricultural markets, are dominated by farming systems that have adopted high use of pesticide, fertilizer, irrigation, and agricultural credit[5]. Thus, in order to obtain an unbiased estimate of the ZBNF programme’s impact, we had to sample from both areas.

At each site, we mapped the outline of the largest continuous area under ZBNF using a handheld GPS and with the help of RySS staff and farmers. We also obtained information on the crops grown, the management practices, and the types of farming practiced in the wider region, and recorded landscape features, such as

remnant native trees and riparian buffers. We had to exclude many sites as the area under ZBNF was smaller than we were initially informed. We then visualised the remaining 13 sites in QGIS using Google Earth imagery and placed a 500m x 500m square within the area under ZBNF. Farmers at those sites transitioned from agrichemical farming to ZBNF between 2016 and 2017.

Agrichemical sites ('squares')

We aimed to identify 13 agrichemical squares that had similar attributes to the ZBNF squares. Hence, we marked farmland areas within approximately 15 km of each ZBNF square that had the same soil type and were similar in elevation, temperature, rainfall, slope and amount of natural habitat patches embedded in the agricultural landscape. They were also within the same area (i.e., 'tribal' or 'plain). We then randomly placed 500 x 500m squares within those areas, again requiring sites to be at least 3 km from the nearest land-use of a different kind. We conducted field visits to enquire about the farming practices employed, and we confirmed with local agricultural scientists and practitioners that the selected sites are indeed likely to capture the full range of management practices and agricultural productivity found across the districts.

Forest sites ('squares')

To identify forest sites, we overlayed Copernicus land-use, Landsat imagery, soil type, elevation, slope, rainfall, temperature, and protected area boundary layers in GEE. We clipped out areas of high (>600 m) elevation and National Parks, excluded forests of less than 100 km^2 and areas less than 3 km from the forest edge, and sought sites with similar attributes of the other variables to the agricultural squares.

As in many parts of the world[19,20], the remaining forests in our study region are predominantly where opportunity costs (i.e. income lost by not farming) are low, e.g., at comparatively higher elevations, further from cities, and on uneven terrain. Thus, a limited number of sites met the above-mentioned biophysical criteria. We randomly placed 25 500 x 500 m squares within suitable areas, whilst ensuring that sites were distributed across the study region and encompassed the three major regional forest types (namely, tropical moist deciduous, tropical semi-evergreen, tropical dry deciduous; see Supplementary Figure 3). We conducted site visits to assess their suitability and accessibility and consulted local ecologists and to confirm our sites were representative of the forest types found in the region.

3. Farmer interviews, yield and profit calculations

With the help of local villagers, we identified the people managing the fields upon which the four points in each square were located and as well as up to two more fields in the square we randomly selected. The interview protocol was informed by discussions with, and was trialled on, farmers and RySS staff. We frequently disaggregated questions into components, for example, for a given task such as fertilizing, we asked how many times per year the field was fertilized, how many days and how many people per day it took, and if the people were paid (and if so, how much). We also asked for yield data over the last three years, whether and how management of the field, yield, and expenditure had changed over the last decade and what the reasons for the observed changes (if any) may be. However, we did not include this information in further analyses due to recall issues. We prepared the questionnaire in English but conducted the interviews in Telugu (the local language). The full set of interview questions are given in Supplementary Table 10. We received

ethical approval from the Cambridge Psychology Research Ethics Committee (approval code PRE.2022.090).

We enquired about the size of each focal field (in acres) and verified the answers on a random subset using a handheld GPS in the field and/or QGIS. In the few instances where self-reported yield or cost values seemed implausible, we followed up using phone calls which resolved the issues. Furthermore, we cross-checked that reported yield values are feasible using district-level government statistics[9] and checked with local vendors that the reported input costs are plausible.

We standardised the yield and cost data into per-acre values, and we used simple crop plant lifecycle models based on the information received by the farmers to estimate average annual costs, yields, and revenues of each crop at each field (following[21]). For annual crops no further calculations were necessary to estimate annual yield. We used the most recent (season 2022/2023) self-reported values. Whilst using the repeat-measures of yield would have allowed us to better account for time-varying confounders, farmers noticeably varied in their ability to recall information older than one year, meaning that a considerable number (if not most) of the estimates would have been unreliable.

For tree crops (cashew and coconut) we used the following formula to estimate the annual average yield (Y) per crop, where m is the number of years the crop is mature, Ym the yield of the crop in a mature year, and i are immature stages with different yields in those years respectively (Yi; where some years will be zero-yielding):

$$Y = \frac{i1Yi1 + i2Yi2 + \cdots + mYm}{i1 + i2 + \cdots + m}$$

In order to obtain a total measure of yield per annum per unit area into a common currency, we converted the estimates of mean net annual yield for each crop into a common unit, namely food energy. Since farmers largely reported the pre-processing harvest, we first subtracted the proportion of the harvested product that is uneaten (husks, peels, etc.) and then converted the remaining mass into GJ/acre to estimate the energetic value of each harvest, i.e., harvest-level productivity (see Table S1). We then calculated the sum of the GJ/hectare values of all crops grown on the same field in a year to obtain an estimate of the total annual yield (GJ/hectare) per field.

In addition to these harvest- and field-level productivity estimates, which we used to estimate the effect of the ZBNF programme on yield and profit, we also estimated square-level productivity where we had to account for the forgone production of native and semi-native vegetation patches embedded in the squares. These patches are small forest fragments (< 0.02 km^2), individual native or naturalised trees, and riparian buffers, and hedges. We estimated the area under these patches, tree crops, and cropland by conducting site visits with a handheld GPS and using QGIS. We calculated the mean of the field-level yield (weighted by field size) for each square for tree and non-tree crops separately and then multiplied these estimates by the area under tree crops and cropland respectively, and then summed the two to obtain estimates of the total annual production (in GJ) per square. We used these square-level productivity estimates to look at the relationships between bird densities and yield, at 25 ha level, for each farming practice.

In order to estimate annual economic profit, we first calculated income using the ten-year mean wholesale state-level prices per unit weight of each crop between 2013-2023 to dampen price fluctuations, where we took the January prices from each year[9] and adjusted them for inflation to 2023 values (using the Consumer Price

Index[22]). We then estimated the per harvest (hectare^{-1}) and subsequently annual revenue (hectare^{-1} year^{-1}) for each field (in the same manner as for food energy).

Costs were composed of input costs (e.g., agrichemicals, ZBNF 'wheels', irrigation, seeds), labour costs (wage and/or family labour), equipment rental and/or purchases (e.g., bullock cart, spade, axe, sprayer, tractor), and equipment maintenance costs (e.g., fuel, electricity), but excluded land tenure costs (as these are external to the farming practice and could bias results if included). In all instances we used the costs as reported by the farmers. The same piece of equipment as well as wage labour may be priced slightly differently in different villages, and access to and ability to process ingredients varies between farmers and regions (e.g., the ingredients and their relative proportion in a given ZBNF 'wheel' can vary between villages according to availability, and the costs of a ZBNF 'wheel' may vary depending on whether it is prepared by the farmers themselves or whether it is purchased from someone else in the village). Whilst this introduces heterogeneity, it represents the information upon which the farmers make agroeconomic decisions and reflects the spectrum of how each farming practice is manifested in the region. We costed family labour at the minimum wage rate (separately for females and males) as reported by each farmer. For equipment, we divided the purchase cost by the expected lifetime of the item (as reported by the farmer) and, where it was shared with other fields, by the total area of the fields combined to obtain a yearly per acre cost value. For fields where not all activities are conducted yearly (e.g., when perennial crops are grown or when land levelling only occurs every five years), we estimated the annual cost by dividing by the number of years between activities or the length of the crop lifecycle as appropriate.

We estimated the mean annual cost per acre per annum for each field which we then subtracted from the revenue to estimate the profit (INR $hectare^{-1}$ $year^{-1}$). Square-level estimates were estimated in the same manner as for annual food energy production.

Notably, irrigation and most agrichemical substances are subsidised, with farmers paying frequently around two-thirds of the market value of the product. We chose to use the subsidised prices (i.e., those paid by the farmers) since we wanted to estimate the impact of the ZBNF programme under the current agri-environmental policy landscape within which farmers are making their management decisions. Notably, any positive impact of ZBNF on profit would be greater if these subsidies were abolished.

4. Bird surveys

Data collection

Surveying in both the winter and summer seasons allowed us to record species mainly vocal during the breeding season as well as winter migrants. It also allowed us to capture fluctuating relative resource availability in the different habitat types and surveying repeatedly across years allowed us to account for time-varying square-level confounders.

We avoided conditions of rain, fog, or high winds. In forest squares, where access to points was often difficult because of thick vegetation, we located and marked each point at least one day in advance and, where necessary, cut an access route, clearing sufficient vegetation at each point to allow us stand and turn unimpeded.

We quantified the proportion of ‘closed’ habitats (i.e., features that impaired detectability such as trees, hedges, and high crops) around each survey point using a handheld GPS during our field visits, and QGIS. For forest points, the proportion equalled one.

We measured the horizontal distance from the point to the location of the centre of each cluster or individual bird when it was first detected, using a laser rangefinder, based on direct distance to the bird(s) and angle of elevation. For individuals detected aurally but not seen, we measured the distance to the tree (or other feature) from which the bird vocalized. We recorded all counts (using a Sony PCM-D100 recorder and a Sennheiser ME66 shotgun microphone) so that uncertain identifications could be checked later with experts and/or reference material consulted.

Data processing

We discarded records of species that point counts do not adequately sample, namely largely aerial and/or transient species (following[23]). Thus, we removed swifts and swallows (*Apus affinis*, *Cecropis daurica*, *Cypsiurus balasiensis*, *Hirundo rustica*, and *Hirundo smithii*), large raptors (*Accipiter badius*, *Circaetus gallicus*, *Circus aeruginosus*, *Elanus caeruleus*, *Falco chicquera*, *Haliastur indus*, *Milvus migrans*, *Pernis ptilorhynchus*, and *Spilornis cheela*), and oriental pratincoles *Glareola maldivarum* (which only briefly migrated through our study region).

Distance sampling

We examined fitted detection models by visually checking quantile-quantile plots and the shape of the detection function, and we conducted Chi-square, Kolmogorov-Smirnov and Cramer-von-Mises tests. We removed models that resulted in a poor fit

or failed to converge. If a given species or detectability group at a given site had well-fitting habitat type specific models (forest or farmland respectively) then we chose these and selected the one with the lowest AICc from amongst the possible key function-adjustment term combinations (see Methods). If no habitat type-specific detection function resulted in a good fit, then we used AICc to select the model with the lowest AICc amongst all the single species or group specific detection functions (those with and without the proportion of closed habitat as a covariate). In all instances, we chose single over multi-species detection functions if available for a given species. Once we had a complete set of fitted detection functions, we obtained effective detection radii for each model at each point.

5. Statistical matching

Statistical matching is used to identify appropriate controls and is one of the most commonly used impact evaluation methods[24]. Specifically, it entails matching treatment and counterfactual units on predefined measurable characteristics that influence both the likelihood of a unit being subjected to an intervention and the outcome of interest, ultimately aiming to balance the distribution of covariates in the treated and counterfactual groups[24, 25, 26]. In this way, we can be more confident that any observed difference between groups is due to the intervention, rather than other variables. Although we selected our treatment (ZBNF) and control (agrichemical) sites to be similar to one another, there is still the potential for biasing covariates to be present. Matching controls for these.

Because of differences in the scale of outcome variables (yield, profit, and bird diversity), we conducted matching in different ways for each analysis:

Covariate Choices for Matching for Yield Analyses

We conducted matching at the harvest-level. The covariates we matched on prior to assessing ZBNF's impact on yield are described in Supplementary Table 2. We did not match on whether the site was within a 'tribal' or 'plain' area as this was already exactly balanced between the two farming practices through site selection (see Supplementary Information 2). As robustness checks, we conducted additional analyses for the 'plain' and 'tribal' farmers separately (as these could represent different counterfactual systems) and only using rice harvest data from the main ('kharif') harvesting season (see Supplementary Information 6; Supplementary Figures 9-12).

Since most farmers did not know the exact variety of crop planted, we could not include it as a matching covariate. However, all farmers interviewed obtain seeds in the same manner: they buy new seeds from the government on a decadal basis and in between that use seeds from the previous year's harvest. Each of the 'paired' ZBNF and agrichemical squares farmers obtained new seeds from the government in the same year, and we thus believe it valid to assume that they are the same variety.

Covariate Choices for Matching for Profit Analyses

Prior to assessing ZBNF's impact on profit, we conducted matching at the field-level and only matched on agroecological suitability (Supplementary Figure 7). This is because many potential confounders are captured by the way we estimated profit. We used the same crop price indices for all samples (rather than farmer-reported prices; see Supplementary Information 3), and the crops grown are on average the same between ZBNF and agrichemical farmers (i.e. 'crop type' was balanced, see Supplementary Figure 6). Nonetheless, to ensure the robustness of our conclusions,

we conducted an additional analysis where we matched on the travel time to the nearest city, the percentage of vegetation patches, the number of harvests grown per year at the given field, whether at least one of the harvests was irrigated, and ownership status (owner or tenure farmer; see Supplementary Figure 9). Our conclusions remained the same.

Covariate Choices for Matching for Bird Diversity Analyses

We matched, at the point-level, on elevation, average annual temperature, and the percentage cover of (semi-)native vegetation patches in each square (see Supplementary Figure 8). These covariates may affect the likelihood of adopting ZBNF as well as the abundance and diversity of birds.

Matching Algorithms

There are multiple algorithms that can perform matching. We tested the three most common: nearest-neighbour[27], full[28], and genetic matching[29, 30], and evaluated performance under each. Nearest neighbour matching is one of the most common algorithms. It selects for each treatment unit at least one control unit with the shortest distance values between these units[27,31]. Full matching uses all available units in the data by forming subgroups containing one treated unit and one or more control units (or vice versa) and assigning weights based on subclass membership which are then used to estimate a weighted treatment effect[32]. Full matching is particularly well suited for analysing data sets with similar number of treatment and control units[24] which applies to our data. Genetic matching entails iterative searches to maximise the balance of covariates between treatment and control and it is based on a generalization of the Mahalanobis distance which additionally involves a weight matrix[33]. Genetic matching involves some additional complexities. As recommended

by Diamond and Sekhon[33] we first generated a propensity score based on the above-described covariates and then conducted genetic matching on these covariates as well as on the propensity score. However, due to criticism of this approach[34], we also conducted genetic matching without the propensity score. Since balance may be easier to achieve this way, we also conducted genetic matching where we only included the most important covariates (agroecological suitability, habitat patches, and crop type) in the calculation of distance and weights, but still optimised balance on all covariates.

Distance Metrics

Matching algorithms proceed by measuring the distance between covariates of treatment (ZBNF) and control (agrichemical) groups (the greater the distance of a covariate, the greater the difference between the groups). We tested two methods of measuring distance: Mahalanobis and Propensity Score distance. Mahalanobis distance calculates how many standard deviations a unit is from the mean of other units[35], whereas propensity score combines all covariates into a single distance measure which then estimates the probability of units receiving the treatment[36]. Whilst propensity score matching is the most commonly used method, it has recently received criticism[34], with examples of Mahalanobis outperforming it (e.g.[37]).

Matching Runs

For full robustness, we conducted matching using the two distance metrics, for each of the three algorithms (full, nearest neighbour and genetic). For all distance metric-matching algorithm combinations (apart from full matching), we conducted matching both with and without replacement, the former meaning that controls can be reused and matched to multiple treatment units which tends to yield better balance[38]. Since

we had data on more ZBNF (i.e., treatment) than agrichemical (i.e., control) harvests (115 and 87 respectively) and fields (66 and 55 respectively), we expected matching with replacement to perform better. Supplementary Table 3 provides more details on the different matching runs conducted.

Notably, we employed matching ex-post, i.e., we used it after conducting field surveys to assess covariate balance and if needed to discard or weigh sampling units. We did not use it prior to that because some of the matching covariates were only collected as part of our field surveys and because we predominately matched at the sub-square level. Furthermore, by seeking to select sites of the same soil type and similar climate and terrain attributes we ultimately replicated the theory underpinning matching (i.e., accounting for observable confounders), albeit without the analytical rigour. For most analyses we did not match directly on the above attributes, but instead on agroecological suitability because the former are encompassed in the latter and matching covariates should be kept to a minimum to ensure that suitable matches can be found[24].

In all instances, the estimand targeted was the average treatment effect in the treated (ATT; i.e., the effect of transitioning to ZBNF for the farmers that did so). The ZBNF programme was not randomly rolled out, but farmers choose whether to adopt ZBNF which meant that targeting the average treatment effect in the population (ATE) was not possible[39].

Unobservable confounders

Matching is unlikely to remove all bias and there is always the possibility of unobservable confounding factors[29,40]. If an unaccounted-for variable caused ZNBF to be rolled out in areas where yield/profit, or bird diversity, was already higher, this

could reduce or nullify our conclusions about the programme's impact. The most likely confounders that we weren't able to control for are the complex psychosocial characteristics of the farmers, including their perceptions, attitudes, and social norms. It is possible that a certain profile of farmers was already more likely to manage farms at higher yield/profit, or in ways better for bird diversity, and that these same farmers were the ones to enrol in ZBNF. Having pairs of ZBNF and agrichemical sites in close proximity increases the likelihood that farmers from both groups are influenced by similar cultural and social norms, and have similar psychosocial characteristics, but it does not guarantee it.

However, we believe that such unobservable confounders, if present, are unlikely to be sufficiently strong to change our conclusion regarding the effect of the ZBNF programme on yield, profit, and bird biodiversity outcomes. The reasons for this belief are as follows:

Regarding yield and profit, the ZBNF programme aims to convert all farmers in AP to ZBNF, irrespective of agroclimatic zone, crop type, and cultural and socioeconomic background of the farmers (see above). Farmers in all districts and sub-districts ('*mandals*') in the state have been exposed to the agricultural extension services of the ZBNF programme[12]. Thus, the ZBNF programme was not selectively rolled out in areas that were high-yielding or unusually profitable, and when selecting sites for our study, we ensured to sample from varied socioeconomic and bioclimatic regions, with paired ZBNF and agrichemical sites (see above). Villages where many farmers chose to adopt ZBNF early on (which we needed for our study; see above), may have different social and economic dynamics than villages that did not. However, RySS staff kept long-term records of yield, including prior to the ZBNF adoption, in

some of these villages, and found it to be similar to what agrichemical farmers anecdotally reported.

Regarding bird biodiversity outcomes, this is not *per* se the main objective of ZBNF, which is instead to improve agrarian livelihoods via improving agricultural productivity and reducing costs. As a result, the programme was not preferentially targeted to regions already richer in avian biodiversity. From the extensive interviews we conducted, and time spent in the region, we consider it highly unlikely that farmers that transitioned to ZBNF had more positive attitudes towards biodiversity conservation and were engaging in activities that benefit farmland birds prior to adopting ZBNF (or at least not more so than farmers that did not adopt ZBNF).

In an ideal world, we would conduct a sensitivity analysis to quantify how large unobservable confounders would have to be in order for them to change the conclusions of our study. However, current tools, such as the R package *sensmakr*[41], are only operationalizable for simple frequentist models, and we could not apply this to the complex Bayesian models used in our analysis.

6. ZBNF impact on yield: robustness checks

We initially fitted a model with a more complex nested random effect structure where field was nested within square, but this model was overfitted. Hence, we only included square as a random term in the main model. This meant that the non-independence of repeat harvests of the same fields across seasons (which was the case for 47.1% ZBNF and 51.7% agrichemical fields) was not entirely accounted for. However, fields are managed fundamentally differently in the winter (‘kharif’) and summer (‘rabi’) harvesting seasons (see Supplementary Figure 1). The winter crop, which is almost exclusively rice, represents the main crop. It is economically and

culturally much more important to the farmers, often involving high labour and resource inputs. In contrast, only around half of the farmers grow a summer crop which is seen as a bonus and is largely for their own consumption and/or to improve soil health. It usually simply involves throwing seeds of various legume varieties (usually ‘blackgram’ or ‘greengram’) with little inputs and labour involved.

By matching on crop type, on whether the harvest was rainfed or irrigated, and on the proportion of the harvest sold we ensured that harvest season level characteristics would not confound our results. Nonetheless, we also repeated the main analyses but only with the data from the main (‘kharif’) harvest of rice. We matched on the same covariates as we did in our main analyses apart from crop type (since we only sampled rice harvests) and travel time to the nearest city (since it was correlated with agroecological suitability). Four matching runs achieved balance with a sufficiently large sample size (Supplementary Figure 10) and all post-matching analyses suggest that ZBNF does not affect ‘kharif’ rice productivity (Supplementary Table 4).

With the expectation that the impact of ZBNF hinges on the agricultural system it is replacing, we subdivided our data into harvests from the ‘plain’ and ‘tribal’ area (see Supplementary Information 2). Two matching runs each met our balance and sample size criteria (Supplementary Figure 11 & 12) and found that ZBNF does neither affect productivity in the ‘plain’ nor ‘tribal’ areas (Supplementary Table 4).

Lastly, our results remained the same when we included the outliers (four tapioca harvests) in the model (Supplementary Table 4).

7. Trade-offs between productivity and bird conservation: additional notes

In addition to the model described in the main text, we also fitted a model that included the percentage cover of native and semi-native vegetation in a given square as a fixed term. However, this model failed to converge (i.e., Rhat values were > 1). Nonetheless, the effect of vegetation cover on productivity is internalised in the main model since any forgone food production associated with these vegetation patches has been accounted for when calculating square-level yield and profit (see Supplementary Information 3).

Our analysis of the impact of the ZBNF programme on bird densities aims to disentangle causal relationships by using statistical matching to control for confounding factors that may bias our estimates of ZBNF. We did not include square-level productivity in these analyses, because the relationship between the covariates and the outcome cannot be validly interpreted post matching (as the covariates were balanced between groups; termed the Table 2 fallacy[42]).

The outcomes of our main model examining density-productivity relationships should not be interpreted as causal effects of agricultural intensification on bird densities, but, instead, an assessment of the trade-offs between increasing agricultural productivity at the landscape-scale (a product of field-level management and landscape-level vegetation cover) and bird species densities. The square-level percentage cover of native and semi-native vegetation was similarly low for both farming systems (Wilcoxon–Mann–Whitney test, $P = 0.118$), where 84% of all squares had vegetation covers of less than 15%. Thus, square-level productivity and thus productivity-density relationships are likely to be largely, but not exclusively, driven by field-level management.

8. Bray-Curtis dissimilarity index at the square-level

We repeated our analysis of the community similarity of ZBNF and of agrichemical systems to natural forests at the square-level, meaning that we calculated the Bray-Curtis dissimilarity index between each ZBNF and each forest square as well as between each agrichemical and each forest square. Our model was similar to that described in Formula 4 (see Methods), but without the nested random effect structure (of points nested within squares). The results were similar: ZBNF improved overall community integrity by 3.9% (2.86 to 4.96%) relative to agrichemical farming, where the Bray-Curtis dissimilarity index between forests and ZBNF was 0.89 [95% CI: 0.88 to 0.90)] and between forests and agrichemical systems 0.93 [0.92 to 0.93]. That the Bray-Curtis indices were estimated to be slightly lower at the square-level than at the point-level (i.e. the forest and agricultural communities estimated to be more similar) could be due to a relatively high species turnover resulting in a high dissimilarity of points within a given square.

References

1. Bharucha, Z. P., Mitjans S. B., and Pretty, J. Towards redesign at scale through zero budget natural farming in Andhra Pradesh, India. *International Journal of Agricultural Sustainability* 18.1, 1-20 (2020)
2. Smith, J., Yeluripati, J., Smith, P. and Nayak, D.R. Potential yield challenges to scale-up of zero budget natural farming. *Nature sustainability 3*(3), 247-252 (2020)
3. Khadse, A. and Rosset, P.M. Zero Budget Natural Farming in India–from inception to institutionalization. *Agroecology and Sustainable Food Systems*, *43*(7-8), 848-871 (2019)

4. Khadse, A., Rosset, P.M., Morales, H. and Ferguson, B.G. Taking agroecology to scale: The zero budget natural farming peasant movement in Karnataka, India. *The Journal of Peasant Studies 45*(1), 192-219 (2018)
5. GIST Impact Report. *Natural Farming Through a Wide-Angle Lens: True Cost Accounting Study of Community Managed Natural Farming in Andhra Pradesh, India.* GIST Impact, Switzerland and India (2023)
6. Duddigan, S. et al. Natural farming improves crop yield in SE India when compared to conventional or organic systems by enhancing soil quality. *Agronomy for Sustainable Development* 43.2, 1-15 (2023)
7. Sitharaman, N. Budget 2019–2020 speech. *India Ministry of Finance* (5 July 2019); https://www.indiabudget.gov.in/budgetspeech.php
8. Sutherland, W. J., et al. A 2021 horizon scan of emerging global biological conservation issues. *Trends in Ecology & Evolution* 36.1, 87-97 (2021)
9. ICRISAT–TCI. *District Level Data for India (DLD).* http://data.icrisat.org/dld/ (2020)
10. Ray, D. K., et al. Recent patterns of crop yield growth and stagnation. *Nature communications* 3.1, 1293 (2012)
11. Veluguri, D., et al. Political analysis of the adoption of the Zero-Budget natural farming program in Andhra Pradesh, India. *Agroecology and Sustainable Food Systems* 45.6, 907-930 (2021)
12. *Zero Budget Natural Farming* http://apzbnf.in (RySS, Government of Andhra Pradesh, 2024).
13. Buchhorn, M. et al. Copernicus global land cover layers—collection 2. *Remote Sensing* 12.6, 1044 (2020)

14. Pereponova, A., et al. Use of the term “landscape” in sustainable agriculture research: a literature review. *Heliyon* 9,11, e22173 (2023)
15. Farr, T. G., et al. The shuttle radar topography mission. *Reviews of geophysics* 45.2 (2007)
16. Funk, C. et al. The climate hazards infrared precipitation with stations—a new environmental record for monitoring extremes. *Scientific data* 2.1, 1-21 (2015)
17. Hersbach, H., et al. The ERA5 global reanalysis. *Quarterly Journal of the Royal Meteorological Society* 146.730, 1999-2049 (2020)
18. Nachtergaele, F. et al. The harmonized world soil database. *Proceedings of the 19th World Congress of Soil Science, Soil Solutions for a Changing World, 1-6* (2010)
19. Joppa, L.N. and Pfaff, A., High and far: biases in the location of protected areas. *PloS one*, *4*(12), p.e8273 (2009)
20. Robalino, J.A. and Pfaff, A. Contagious development: Neighbor interactions in deforestation. *Journal of Development Economics*, *97*(2), pp.427-436 (2012)
21. Phalan, B., Onial, M., Balmford, A. and Green, R.E. Reconciling food production and biodiversity conservation: land sharing and land sparing compared. *Science*, 333, 1289-1291 (2011)
22. International Monetary Fund. *Consumer Price Index* https://data.imf.org/?sk=4ffb52b2-3653-409a-b471-d47b46d904b5 (2024)
23. Gilroy, James J., et al. EDITOR'S CHOICE: Surrounding habitats mediate the trade-off between land-sharing and land-sparing agriculture in the tropics. *Journal of applied ecology* 51.5, 1337-1346 (2014)
24. Schleicher, J. et al. Statistical matching for conservation science. *Conservation Biology* 34.3, 538-549 (2020)

25. Morgan, S.L. and Harding, D.J. Matching estimators of causal effects: Prospects and pitfalls in theory and practice. *Sociological methods & research*, *35*(1), 3-60 (2006)
26. Stuart, E. A. Matching methods for causal inference: A review and a look forward." *Statistical science: a review journal of the Institute of Mathematical Statistics* 25.1, 1 (2010)
27. Althauser, R.P. and Rubin, D., 1970. The computerized construction of a matched sample. *American Journal of Sociology*, *76*(2), 325-346 (1970)
28. Rosenbaum, P.R. *Overt bias in observational studies* Springer New York (2002)
29. Ferraro, P.J. and Simorangkir, R. Conditional cash transfers to alleviate poverty also reduced deforestation in Indonesia. *Science Advances*, *6*(24), p.eaaz1298 (2020)
30. Ribas, L.G., Pressey, R.L. and Bini, L.M. Estimating counterfactuals for evaluation of ecological and conservation impact: an introduction to matching methods. *Biological Reviews*, *96*(4), 1186-1204 (2021)
31. Rubin, D.B., Bias reduction using Mahalanobis-metric matching. *Biometrics* 293-298 (1980)
32. Hansen, B.B. Full matching in an observational study of coaching for the SAT. *Journal of the American Statistical Association*, *99*(467), 609-618 (2004)
33. Diamond, A. and Sekhon, J.S. Genetic matching for estimating causal effects: A general multivariate matching method for achieving balance in observational studies. *Review of Economics and Statistics*, *95*(3), 932-945 (2013)

34. King, G. and Nielsen, R. Why propensity scores should not be used for matching. *Political analysis*, *27*(4), 435-454 (2019)
35. Legendre, P. and Legendre, L., 2012. *Numerical ecology* (Vol. 24). Elsevier. (2012)
36. Rosenbaum, P.R. and Rubin, D.B. The central role of the propensity score in observational studies for causal effects. *Biometrika*, *70*(1), 41-55 (1983)
37. Wauchope, H. S., et al. Protected areas have a mixed impact on waterbirds, but management helps. *Nature* 605.7908, 103-107 (2022)
38. Austin, P.C. The performance of different propensity score methods for estimating marginal hazard ratios. *Statistics in medicine*, *32*(16), 2837-2849 (2013)
39. Greifer, N. and Stuart, E.A. Choosing the estimand when matching or weighting in observational studies. *arXiv e-prints*, pp.arXiv-2106 (2021)
40. Rasolofoson, R.A. Statistical matching for conservation science revisited: response to Schleicher et al. 2020. *Conservation biology*, *36*(6) (2022)
41. Cinelli, C. and Hazlett, C., 2020. Making sense of sensitivity: Extending omitted variable bias. Journal of the Royal Statistical Society Series B: Statistical Methodology, 82(1), pp.39-67.
42. Westreich, D. and Greenland, S. The table 2 fallacy: presenting and interpreting confounder and modifier coefficients. *American journal of epidemiology*, *177*(4), 292-298 (2013)

Supplementary Figures

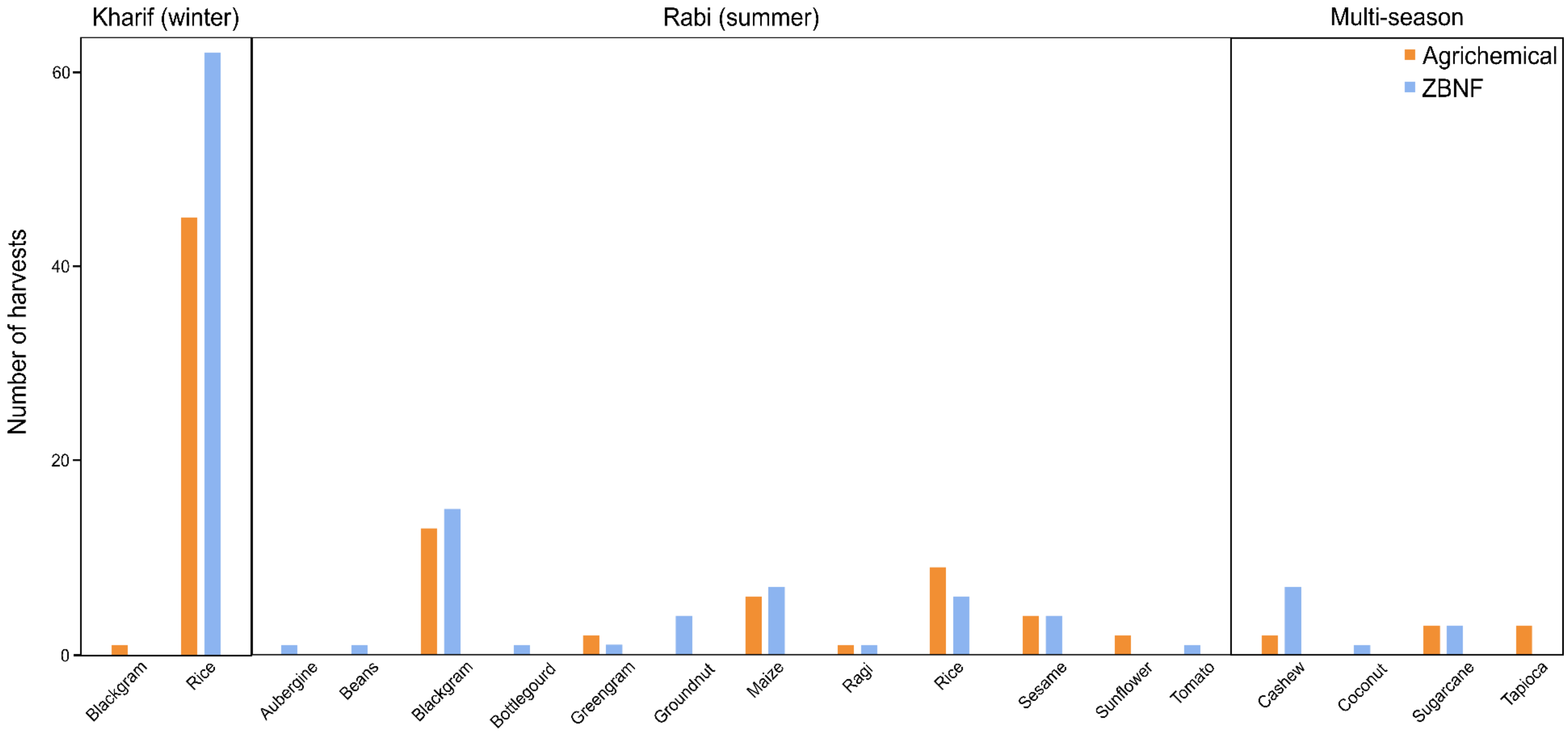

Supplementary Figure 1. Total number of harvests of each crop in each harvesting season under agrichemical farming (orange) and Zero Budget Natural Farming (blue) summed across all fields in our study.

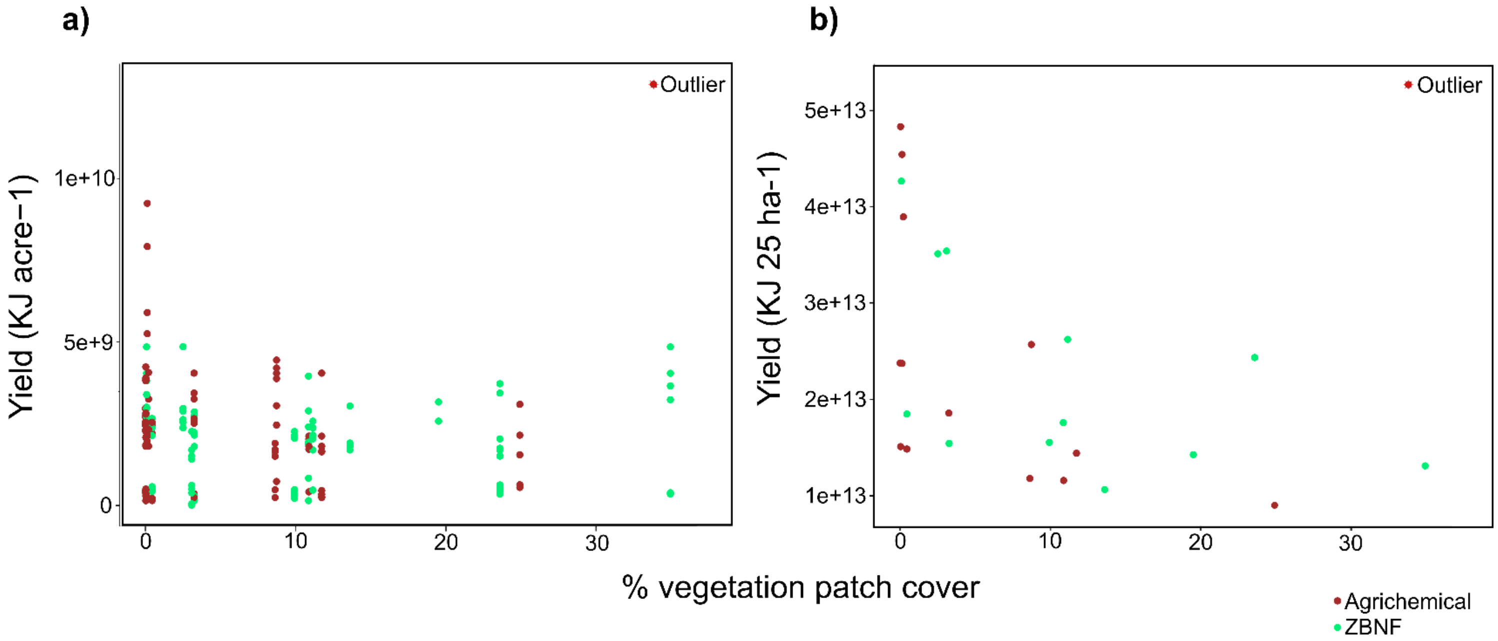

Supplementary Figure 2. Raw a) harvest-level and b) landscape-level yield data along the vegetation patch cover continuum. The colours correspond to the two different farming practices: agrichemical (brown) and Zero Budget Natural Farming (green). Outliers (tapioca harvests and one agrichemical landscape within which tapioca was grown) were removed from our analyses.

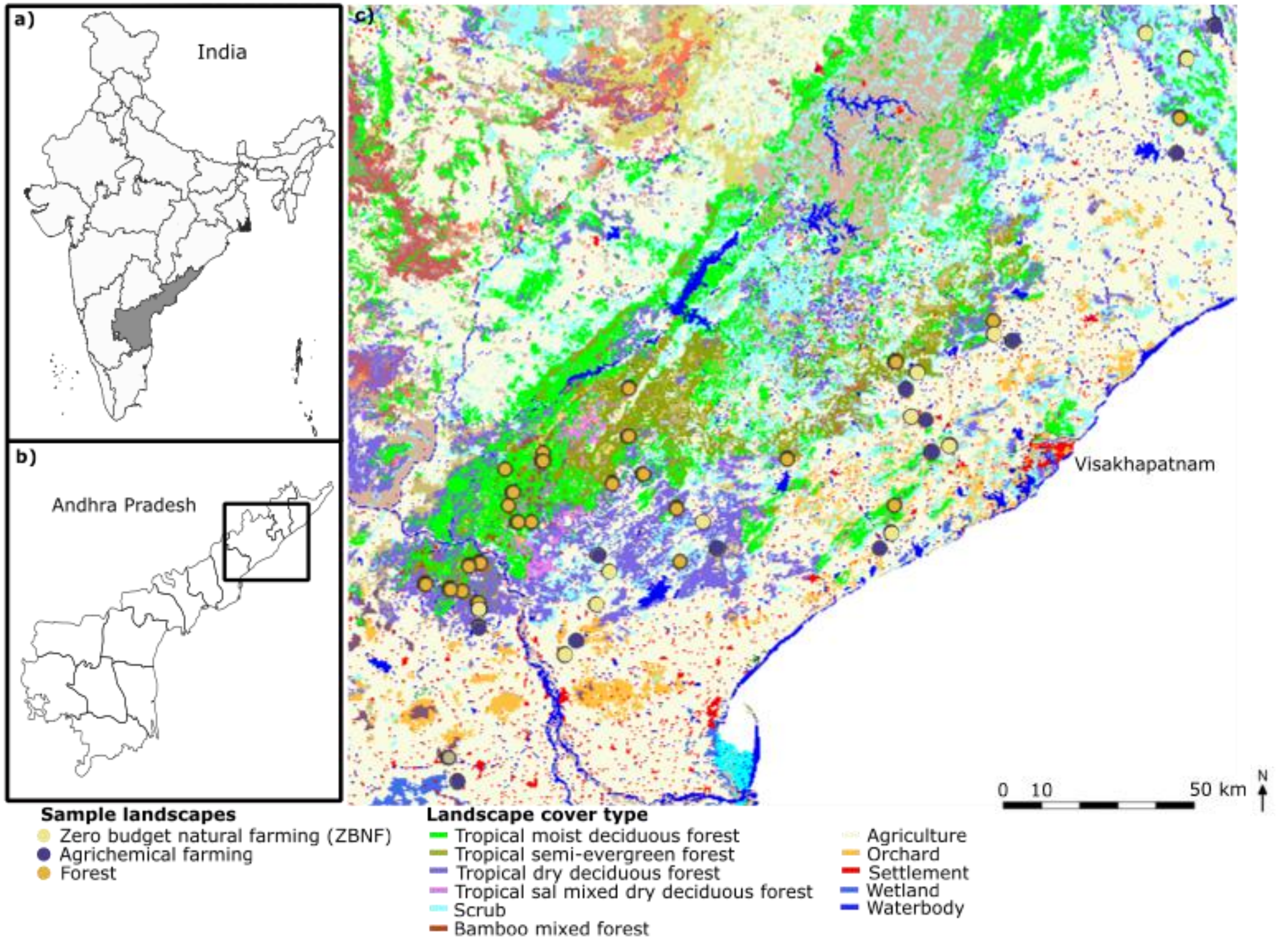

Supplementary Figure 3. Overview of study locations. a) State boundaries within India with Andhra Pradesh shaded in. b) Map of the districts within Andhra Pradesh with the study region highlighted. c) Study area with different land cover types (from Roy et al., 2015) and the study sites depicted.

Overarching question	Sub-question	Data	Data preperation	Final model
1) Effect of ZBNF on yield and profit	1a) Effect of ZBNF on yield	Interviews with farmers to get data on yield of each crop harvest	Matching between ZBNF and agrichemical data, at harvest level	Gaussian linear mixed model, g-computation
	1b) Effect of ZBNF on profit	Interviews with farmers to get data on annual profit per field	Matching between ZBNF and agrichemical data, at field level	Gaussian linear mixed model, g-computation
2) Effect of ZBNF on birds		Birds surveys (point-count based distance sampling) in ZBNF and agrichemical landscapes	1) Detection functions to obtain the 'effective area surveyed' for each species at each point<br>2) Matching between ZBNF and agrichemical data, at point count location level	Zero inflated Poisson count model (Model Formula 1)
3) Trade-offs in ZBNF and agrichemical systems	3a) Density-yield trade-offs in ZBNF and agrichemical systems	Yield and bird survey data as above, plus bird surveys in forest landscapes	Detection functions to obtain the 'effective area surveyed' for each species at each point	Zero inflated Poisson count model (Model Formula 2)
	3b) Density-yield trade-offs in ZBNF and agrichemical systems	Profit and bird survey data as above, plus bird surveys in forest landscapes	Detection functions to obtain the 'effective area surveyed' for each species at each point	Zero inflated Poisson count model (profit-equivalent to Model Formula 2)
4) Comparison to forests	4a) Densities of species of conservation importance relative to forests	As 3a)		
	4b) Richness of species of conservation importance	Bird survey data in ZBNF, agrichemical, and forest landscapes as above	Averaging of 'effective area surveyed' across all species at a given point	Zero inflated Poisson count model (Model Formula 3)
	4c) Bird community similarity to forests	Bird survey data in ZBNF, agrichemical, and forest landscapes as above	1) Calculation of abundance-based Bray–Curtis similarity index between each forest point and each agricultural point<br>2) Matching between ZBNF and agrichemicaldata, at point count location level	Zero-one inflated beta model (Model Formula 4)

Supplementary Figure 4. Outline of how the field survey data were integrated into the statistical models we conducted to evaluate the key research questions of the study. Model formula numbers refer to those described in the main text.

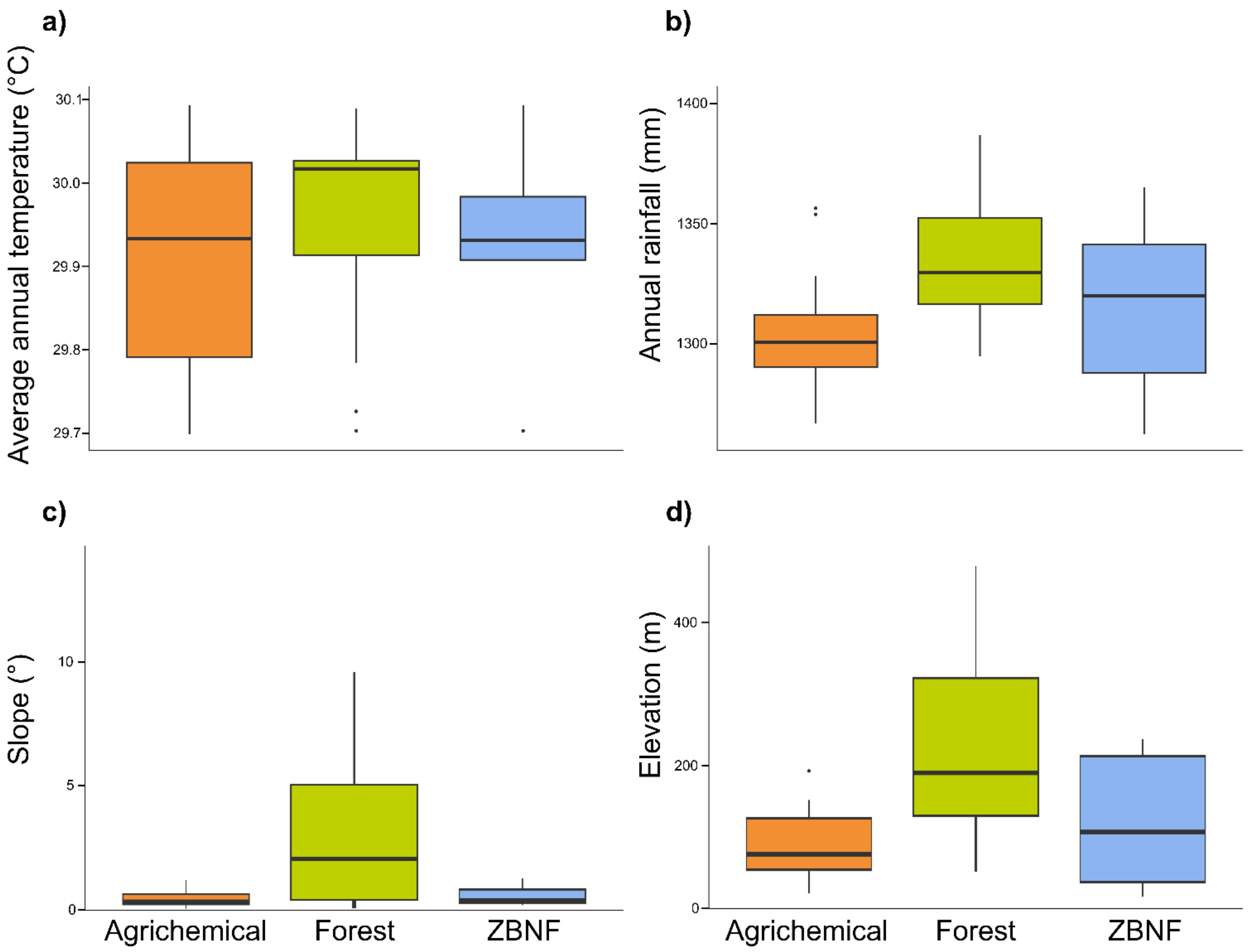

Supplementary Figure 5. Box plots depicting the distribution of four biophysical variables: a) average annual temperature, b) total annual rainfall, c) slope, and d) elevation of agrichemical (orange), forest (green), and ZBNF (blue) landscapes (squares). Each box represents the interquartile range (IQR), with the median indicated by the horizontal line inside the box. Whiskers extend to 1.5 times the IQR.

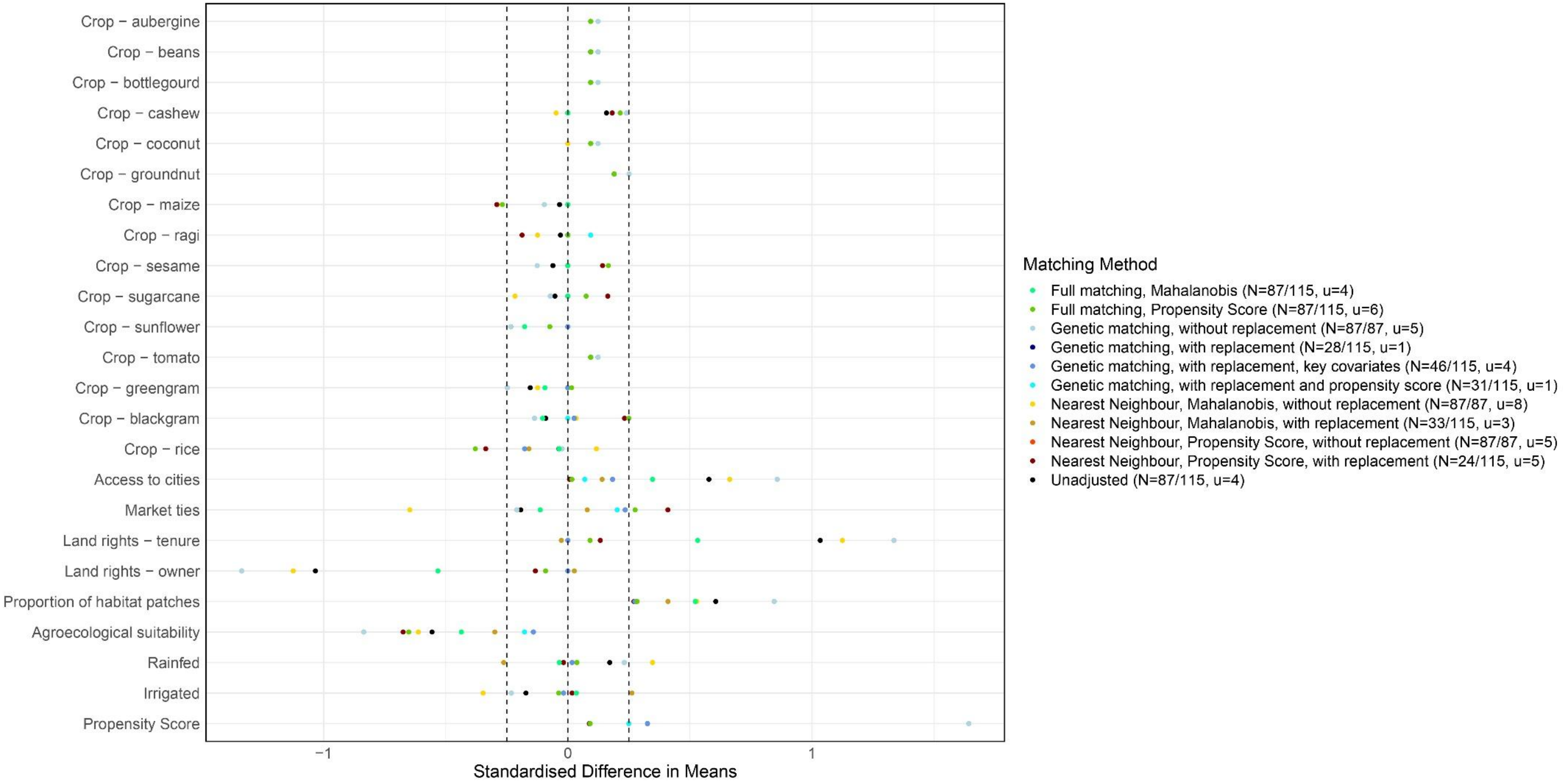

Supplementary Figure 6. Standardised difference in means of matching covariates between ZBNF and agrichemical harvests (for the analysis on yield). The legend describes the different matching runs, with the sample size (N= agrichemical/ZBNF harvests) and the number of covariates with an SDiM > 0.25 (u) in brackets.

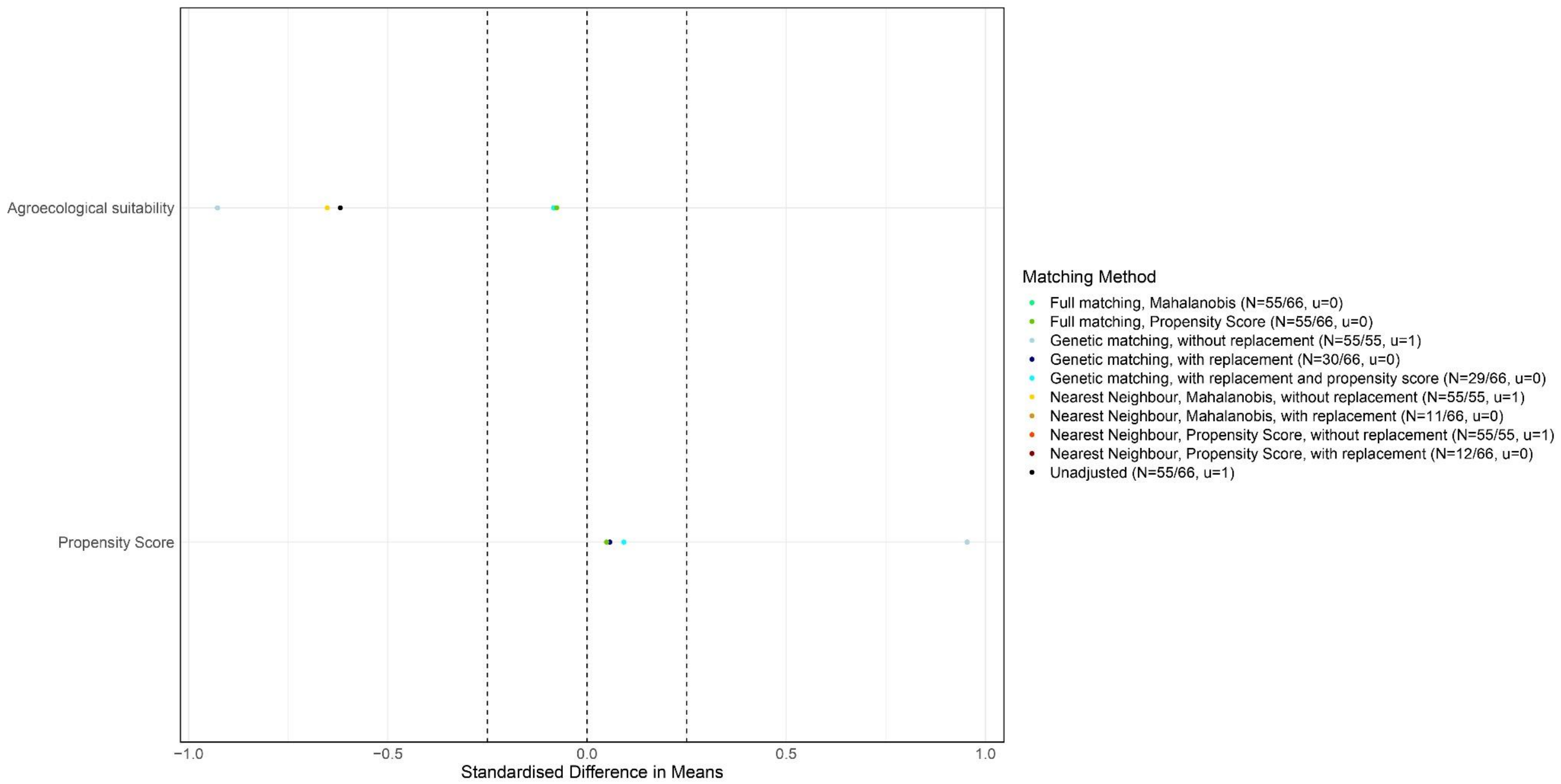

Supplementary Figure 7. Standardised difference in means of matching covariates between ZBNF and agrichemical fields (for the analysis on profit). The legend describes the different matching runs, with the sample size (N= agrichemical/ZBNF harvests) and the number of covariates with an SDiM > 0.25 (u) in brackets.

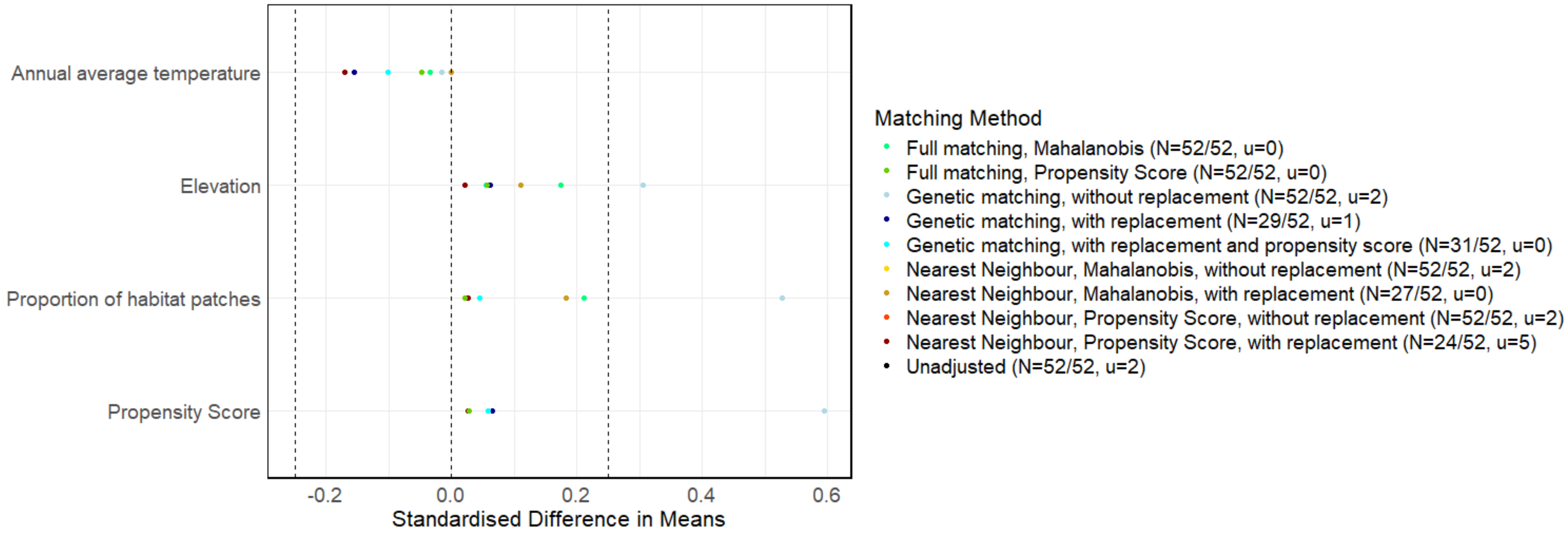

Supplementary Figure 8. Standardised difference in means of matching covariates between ZBNF and agrichemical bird point count locations. The legend describes the different matching runs, with the sample size (N= agrichemical/ZBNF points) and the number of covariates with an SDiM > 0.25 (u) in brackets.

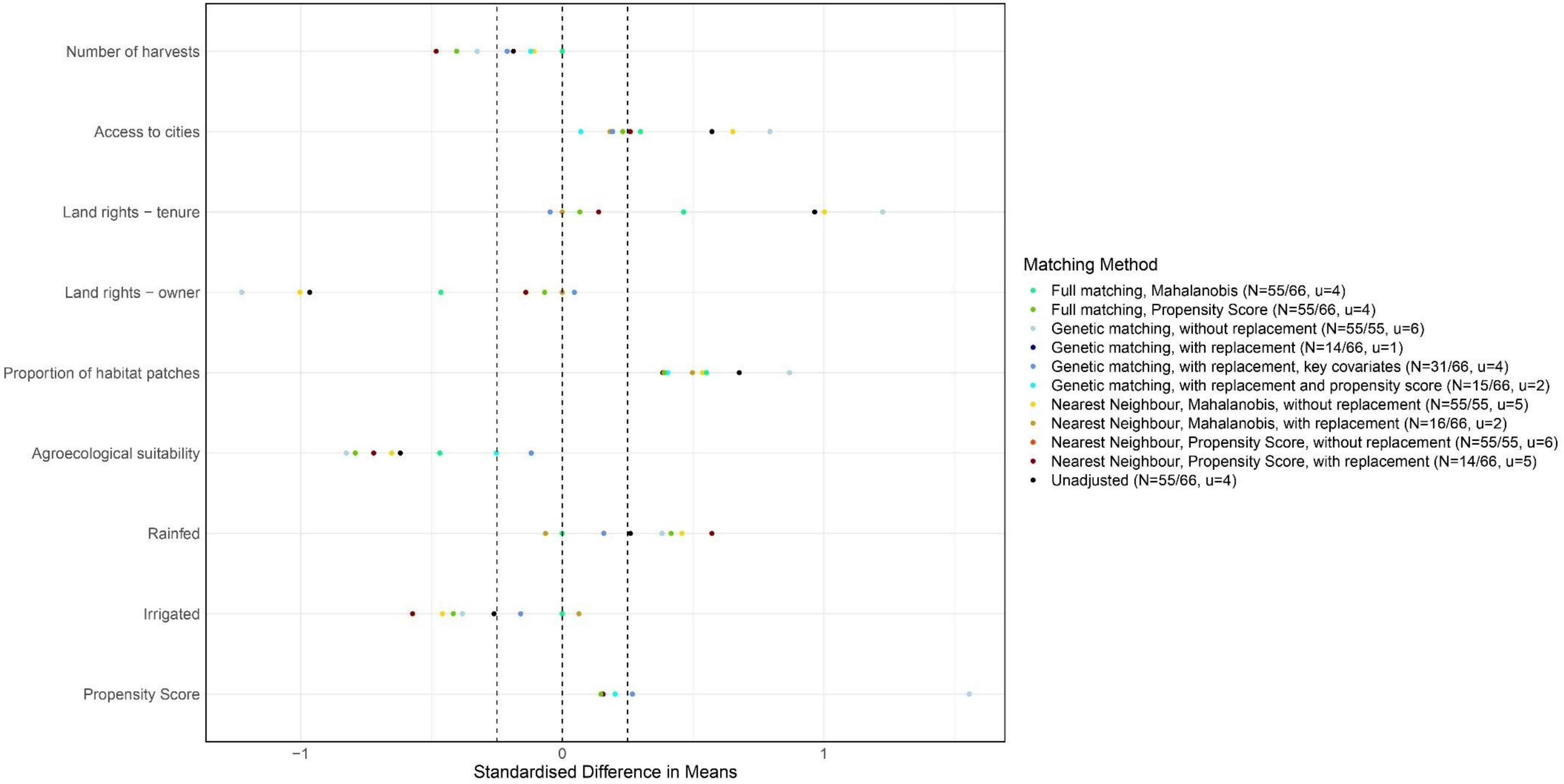

Supplementary Figure 9. Standardised difference in means of matching covariates between ZBNF and agrichemical fields (for the supplementary analysis). The legend describes the different matching runs, with the sample size (N= agrichemical/ZBNF harvests) and the number of covariates with an SDiM > 0.25 (u) in brackets.

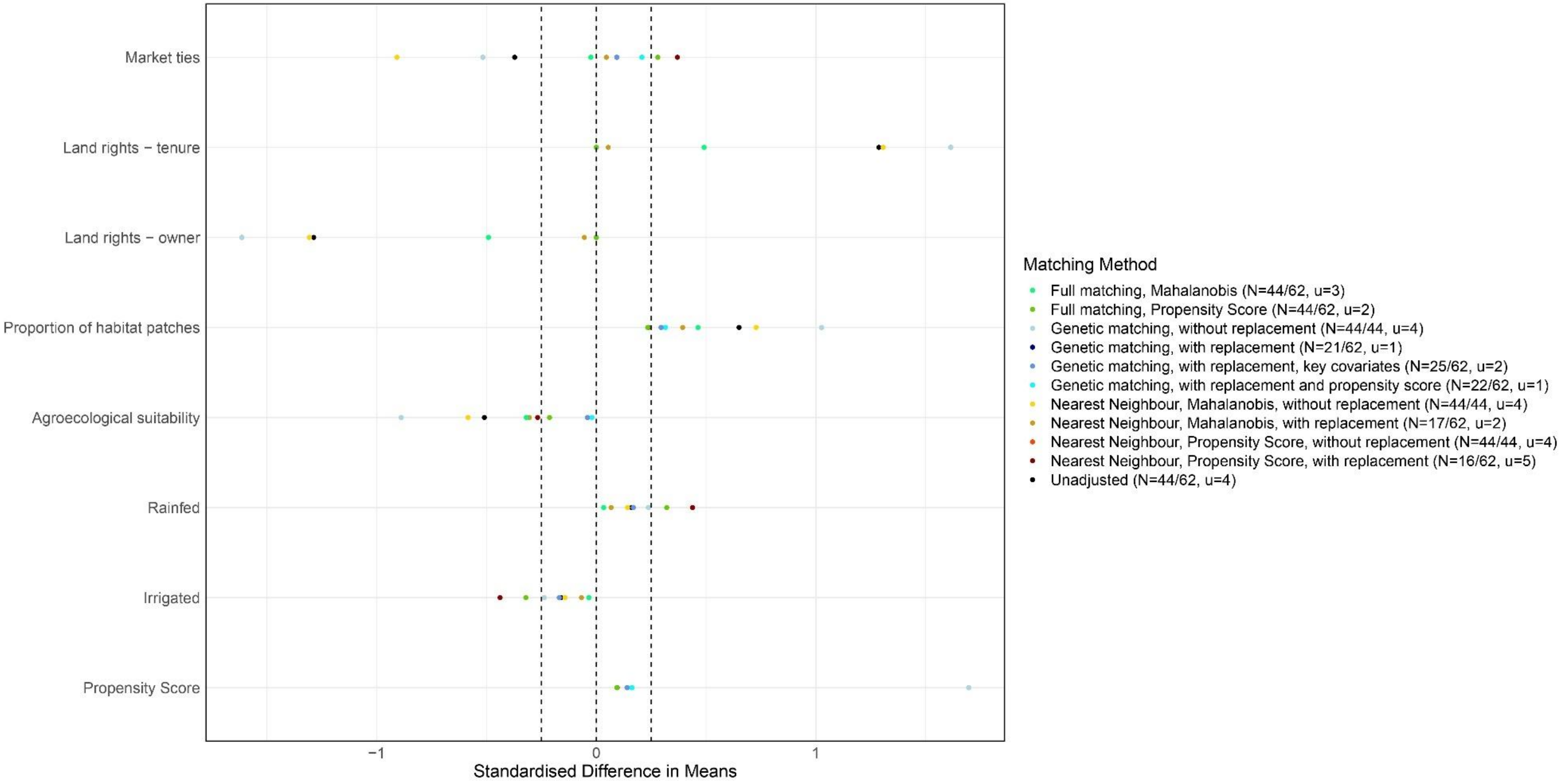

Supplementary Figure 10. Standardised difference in means of matching covariates between ZBNF and agrichemical rice harvests of the main ('kharif') growing season. The legend describes the different matching runs, with the sample size (N= agrichemical/ZBNF harvests) and the number of covariates with an SDiM > 0.25 (u) in brackets.

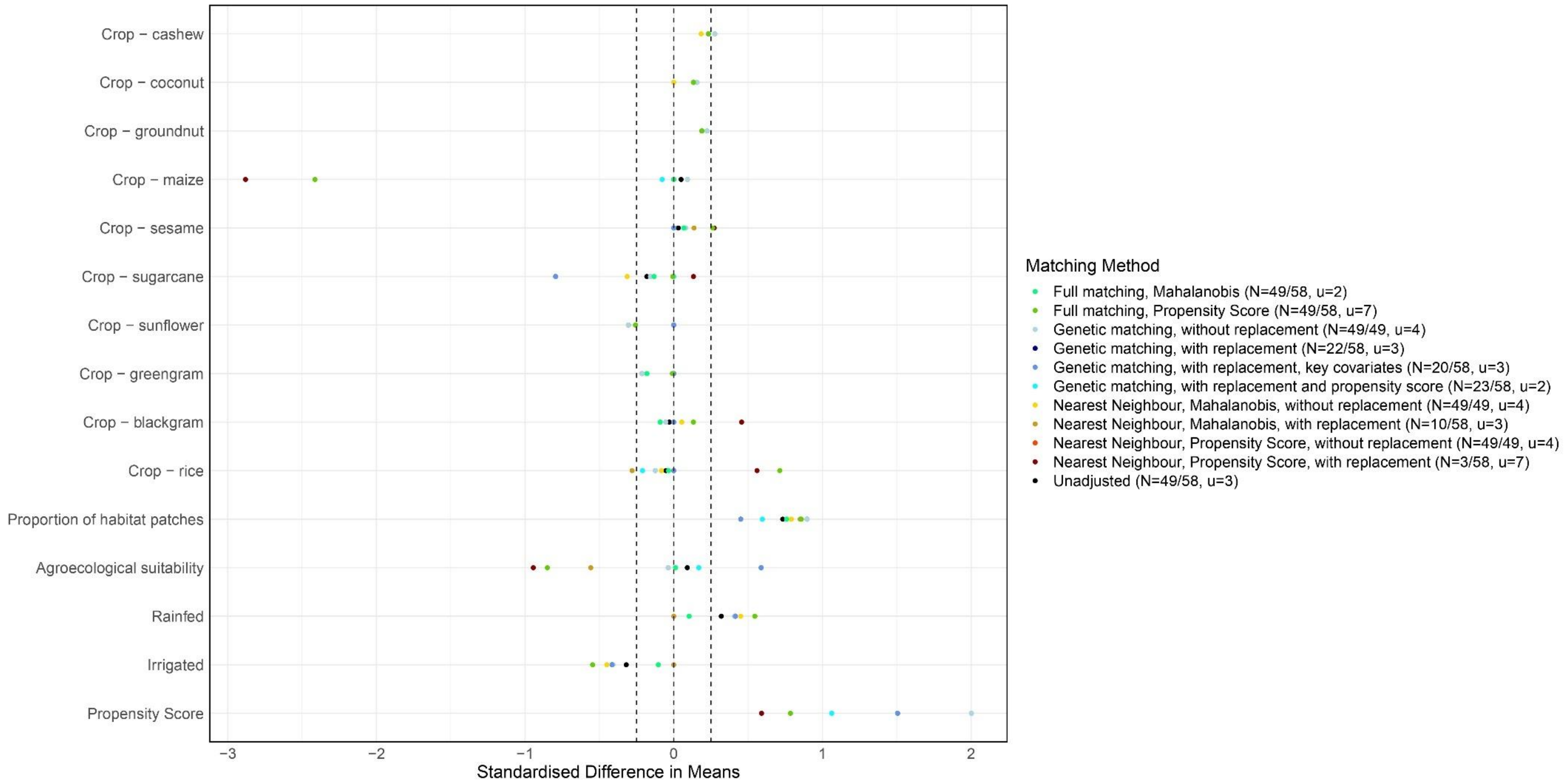

Supplementary Figure 11. Standardised difference in means of matching covariates between ZBNF and agrichemical harvests of the 'plain' area only. The legend describes the different matching runs, with the sample size (N= agrichemical/ZBNF harvests) and the number of covariates with an SDiM > 0.25 (u) in brackets.

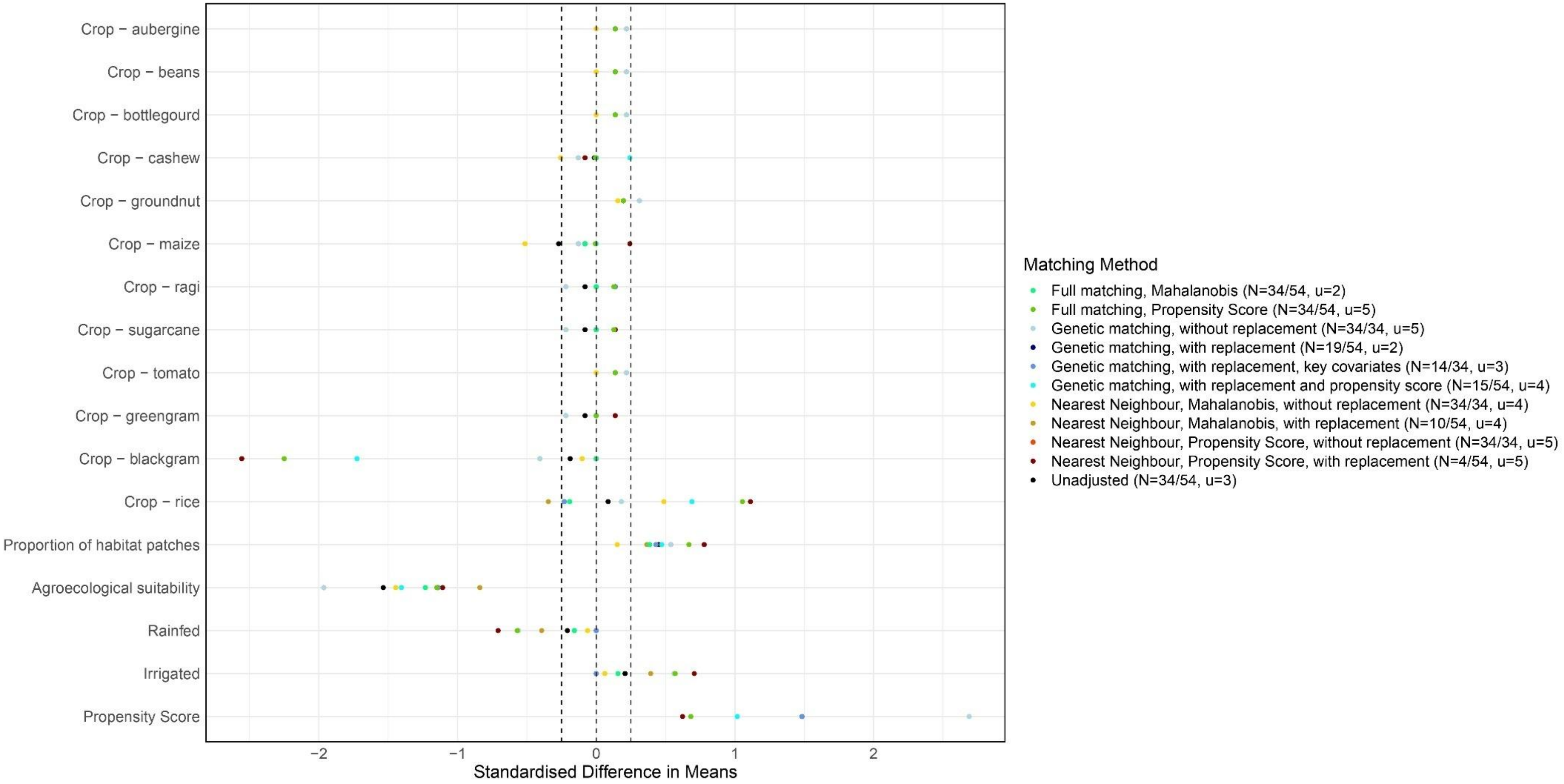

Supplementary Figure 12. Standardised difference in means of matching covariates between ZBNF and agrichemical harvests of the 'tribal' area only. The legend describes the different matching runs, with the sample size (N= agrichemical/ZBNF harvests) and the number of covariates with an SDiM > 0.25 (u) in bracket

Supplementary Tables

Supplementary Table 1. Percentage of harvested mass discarded and food energy per crop. Source: USDA (2019). This represents the inedible percentage of the harvest mass as reported by the farmers and thus minor processing may have already occurred for some crops (e.g., farmers report maize harvest as dried maize removed from the cob).

Crop	Discarded (%)	Energy (KJ/100g)
aubergine	19	104
beans	0	368
blackgram	0	1427
bottlegourd	30	59
cashew	70	2314
coconut	48	1481
greengram	0	1452
groundnut	30	2374
maize	0	1616
ragi	0	1582
rice	25	1506
sesame	0	2397
sugarcane	90	1618
sunflower	46	2445
tapioca	14	1498
tomato	9	74

Supplementary Table 2. Matching covariates when analysing ZBNF's impact on yield

Category, reason for inclusion, and variables	Data source	Data type
**Agricultural (agroecological) suitability.** A measure of how suitable the land is for agriculture (based on soil, terrain, and climatic conditions). This influences crop yield (as high agricultural has a positive effect on yield) as well as the management decisions made by the farmers. We expect that farmers may invest less in land of low agricultural suitability and may be more willing to take risks, for example by adopting ZBNF.	Global Agro-Ecological Zones (GAEZ v3.0) (FAO/IIASA, 2012); https://gaez.fao.org/; 5 arc-minute resolution	Continuous
**Proportion of small-scale (semi-)native vegetation cover**. The proportion of natural and semi-natural vegetation in agricultural landscapes may affect crop yield via ecosystem service provisioning (e.g., pollination, pest control, water holding capacity, shade) and disservices (e.g., pests). We expect that farmers in areas where there is a high proportion of (semi-)native vegetation cover are more likely to adopt ZBNF.	Fieldwork and Google Earth in QGIS	Continuous
**Crop type.** Crops vary in food energy and thus affect yield outcomes. Some crop types may be perceived as more suitable to be managed under a particular farming system than others either due to the biophysical characteristics of the crop or the intended use. For example, farmers may be more willing to adopt ZBNF for crop types that require less intensive management or are less profitable.	Interviews	Categorical
**Travel time to the nearest city of population >50,000.** Farmers closer to cities tend to have greater access to markets, technology, agrichemicals, and other resources. For example, farmers in proximity to urban centres are more likely to adopt new farming technologies (Kumar et al., 2020). They also tend to have larger landholdings and greater economic wealth and spending power. These factors influence the probability of adoption of ZBNF, the exact management practices adopted, as well as yield and profit directly. We expect farmers near cities to be less likely to adopt ZBNF.	Weiss et al., 2018	Continuous
**Proportion of harvest sold.** Agronomic decisions are influenced by whether the crop grown is primarily sold at the market or consumed by the farmers themselves. Time and money invested, the farming practice employed, and yield and profit are all likely to be affected. We expect that farmers selling all or a high proportion of their produce are less likely to adopt ZBNF and more likely make management decisions that involve high financial investment.	Interviews	Continuous

**Land rights.** Whether the farmer owns the land or is a tenant farmer influences the agroeconomic decisions made, especially with regards to long-term sustainability. We expect that tenant farmers are less likely to adopt ZBNF as they bear the transition risks and may not experience any long-term productivity benefits from boosting soil health. Conversely, they may be more likely to engage in management practices aimed at boosting short-term productivity.	Interviews	Categorical
**Irrigation.** Irrigation positively affects agricultural productivity, and it may mediate ZBNF's impact on yield (GIST Impact Report, 2023). It may also negatively affect the likelihood of ZBNF adoption as ZBNF may be perceived as less profitable in irrigated systems.	Interviews	Continuous

Supplementary Table 3. Matching runs conducted and covariates included in post-matching regression analyses. The main matching run and analyses (i.e., focal analyses of the main text) are shaded in grey.

Algorithm	Distance metric	With replacement	Response variable: yield (GJ acre^{-1})			Response variable: profit (INR acre^{-1})		
			**Covariates matched on**	**Covariates optimised balance on**	**Covariates included in post-matching regression analysis**	**Covariates matched on**	**Covariates optimised balance on**	**Covariates included in post-matching regression analysis**
Full matching	Mahalanobis	NA	agroecological suitability, percentage habitat patches, ownership, travel time to a city, crop type	agroecological suitability, percentage habitat patches, ownership, travel time to a city, crop type		agroecological suitability	agroecological suitability	None
	Propensity Score	NA	agroecological suitability, percentage habitat patches, ownership, travel time to a city, crop type	agroecological suitability, percentage habitat patches, ownership, travel time to a city, crop type		agroecological suitability	agroecological suitability	None
Nearest Neighbour	Mahalanobis	Y	agroecological suitability, percentage habitat patches, ownership, travel time to a city, crop type	agroecological suitability, percentage habitat patches, ownership, travel time to a city, crop type	agroecological suitability, habitat patches, irrigation	agroecological suitability	agroecological suitability	
	Mahalanobis	N	agroecological suitability, percentage habitat patches, ownership, travel time to a city, crop type	agroecological suitability, percentage habitat patches, ownership, travel time to a city, crop type		agroecological suitability	agroecological suitability	
	Propensity Score	Y	agroecological suitability, percentage habitat patches, ownership, travel time to a city, crop type	agroecological suitability, percentage habitat patches, ownership,		agroecological suitability	agroecological suitability	

				travel time to a city, crop type				
	Propensity Score	N	agroecological suitability, percentage habitat patches, ownership, travel time to a city, crop type	agroecological suitability, percentage habitat patches, ownership, travel time to a city, crop type		agroecological suitability	agroecological suitability	
Genetic	Generalised Mahalanobis	N	agroecological suitability, percentage habitat patches, ownership, travel time to a city, crop type	agroecological suitability, percentage habitat patches, ownership, travel time to a city, crop type		agroecological suitability	agroecological suitability	
	Generalised Mahalanobis	Y	agroecological suitability, percentage habitat patches, ownership, travel time to a city, crop type	agroecological suitability, percentage habitat patches, ownership, travel time to a city, crop type	habitat patches	agroecological suitability	agroecological suitability	agroecological suitability
	Generalised Mahalanobis	Y	agroecological suitability, percentage habitat patches, ownership, travel time to a city, crop type, propensity score	agroecological suitability, percentage habitat patches, ownership, travel time to a city, crop type, propensity score	habitat patches	agroecological suitability	agroecological suitability	agroecological suitability
	Generalised Mahalanobis	Y	agroecological suitability, percentage habitat patches, crop type	agroecological suitability, percentage habitat patches, ownership, travel time to a city, crop type				

Supplementary Table 4. Estimated impact of ZBNF on yield (GJ hectare^{-1}). Risk ratios (E_{ZBNF} / $E_{Agrichemical}$) with standard errors in parentheses and bias-corrected accelerated (BCa) bootstrap confidence intervals 95% CI in brackets. The main matching run and analyses (i.e., focal analyses of the main text) is shaded in grey.

Matching run	Main analysis	Winter ('kharif') rice harvest only	'Plain' area only	'Tribal' area only	Outliers (tapioca harvests) included
Nearest neighbour matching, Mahalanobis, with replacement	0.994 (0.0360) [0.9407, 1.0885]				0.985 (0.0374) [0.919, 1.072]
Genetic matching, with replacement	0.969 (0.0521) [0.872, 1.083]	1.077 (0.050) [0.958, 1.154]		0.849 (0.087) [0.758, 1.096]	0.999 (0.0892) [0.762, 1.059]
Genetic matching, with replacement, including propensity score	1.015 (0.0539) [0.944, 1.193]	1.098 (0.051) [1.000, 1.191]	0.922 (0.0907) [0.837, 1.207]		1.042 (0.0767) [0.793, 1.091]
Genetic matching, with replacement, matched on key covariates (balance on all)		1.103 (0.067) [0.986, 1.251]			
Full matching, Mahalanobis			1.012 (0.0263) [0.964, 1.066]	0.887 (0.066) [0.776, 1.049]	
Full matching, Propensity Score		1.038 (0.041) [0.960, 1.113]			

Supplementary Table 5. Estimated impact of ZBNF on profit (INR hectare^{-1}). Risk ratios (E_{ZBNF} / $E_{Agrichemical}$) with standard errors in parentheses and bias-corrected accelerated (BCa) bootstrap confidence intervals 95% CI in brackets. The 'seven matching covariates' analysis was matched on agroecological suitability, the travel time to the nearest city, the percentage of vegetation patches, the number of harvests grown per year at the given field, whether at least one of the harvests was irrigated, and ownership status (owner or tenure farmer). The main matching run and analyses (i.e., focal analyses of the main text) is shaded in grey.

Matching run	Main analysis	Seven matching covariates
Full matching, Mahalanobis	2.236 (1.757) [1.631, 3.440]	
Full matching, Propensity Score	2.683 (1.565) [2.300, 5.097]	
Genetic matching, with replacement	2.644 (1.622) [2.135, 5.577]	7.981 (1.726) [3.002, 8.293]
Genetic matching, with replacement, including propensity score	2.024 (8.155) [-4.372, 3.750]	

Supplementary Table 6. Conservation status, guild, habitat preferences, detection group, and presence in different land systems of all species recorded during the study. Information on a species' trophic guild and main habitat used by that species were obtained from AVONET (2022). Species regarded as 'of conservation importance' in our study are those with a State of India's Birds (SoIB) priority status of "Moderate" or "High" and/or classified as threatened with extinction under the IUCN Red List ('Near Threatened' or 'Vulnerable'). The group a given species was in to build detection functions is shown, as well as whether a species- or group-level detection function was used to estimate the effective area surveyed (where 'none' indicates that the group-level detection function was used). Whether we recorded a given species in ZBNF, agrichemical, or forest systems respectively is indicated, as well as whether or not we consider the given species to be disturbance sensitive (based on our knowledge of the region's avifauna).

Species	Trophic guild	Preferred habitat	SoIB priority status	Endemicity	IUCN category	Conservation importance	Own detection group	Detection group	ZBNF	Chemical	Forest	Disturbance sensitive
Acridotheres_fuscus	Omnivore	Woodland	Low	Non-endemic	Least Concern	no	none	myna	no	no	yes	no
calidriotheres_ginginianus	Omnivore	Grassland	High	Indian Subcontinent	Least Concern	yes	none	myna	yes	no	no	no
Acridotheres_tristis	Omnivore	Human Modified	Low	Non-endemic	Least Concern	no	Acridotheres_tristis	myna	yes	yes	no	no
Acrocephalus_dumetorum	Invertivore	Wetland	Low	Non-endemic	Least Concern	no	Acrocephalus_dumetorum	warbler	yes	yes	yes	no
Actitis_hypoleucos	Vertivore	Wetland	Moderate	Non-endemic	Least Concern	yes	none	small_wader_lapwing	yes	yes	no	no
Aegithina_tiphia	Invertivore	Forest	Low	Non-endemic	Least Concern	no	Aegithina_tiphia	arboreal_invertivore	no	yes	yes	no
Aethopyga_siparaja	Nectarivore	Forest	Low	Non-endemic	Least Concern	no	none	sunbirds	no	no	yes	no
streda_gulgula	Omnivore	Grassland	High	Non-endemic	Least Concern	yes	Alauda_gulgula	ground_feeding_passerines	yes	yes	no	yes
Alcedo_atthis	Vertivore	Riverine	Low	Non-endemic	Least Concern	no	none	kingfisher	yes	yes	no	no
Alcedo_hercules	Vertivore	Riverine	Moderate	Non-endemic	Near Threatened	yes	none	kingfisher	yes	no	yes	no
Alcippe_poioicephala	Invertivore	Forest	Moderate	Non-endemic	Least Concern	yes	Alcippe_poioicephala	babbler	no	no	yes	yes
Alexandrinus_krameri	Omnivore	Forest	Low	Non-endemic	Least Concern	no	Alexandrinus_krameri	parakeet	yes	yes	yes	no
Amandava_amandava	Granivore	Shrubland	Low	Non-endemic	Least Concern	no	none	ground_feeding_passerines	no	yes	no	no
Amaurornis_phoenicurus	Vertivore	Wetland	Low	Non-endemic	Least Concern	no	none	large_wader	yes	yes	no	no
Ammomanes_phoenicura	Omnivore	Grassland	High	Indian Subcontinent	Least Concern	yes	none	ground_feeding_passerines	yes	yes	no	yes
Anastomus_oscitans	Vertivore	Wetland	Low	Non-endemic	Least Concern	no	none	large_wader	yes	yes	no	no
Anhinga_melanogaster	Vertivore	Wetland	Low	Non-endemic	Near Threatened	yes	none	large_wader	yes	no	no	no

Anthracoceros_coronatus	Frugivore	Forest	Moderate	Indian Subcontinent	Near Threatened	yes	none	arboreal_frugivore	no	no	yes	no
Anthus_godlewskii	Invertivore	Rock	Low	Non-endemic	Least Concern	no	none	pipit	yes	yes	no	yes
zostus_rufulus	Invertivore	Grassland	Low	Non-endemic	Least Concern	no	Anthus_rufulus	pipit	yes	yes	no	yes
Arachnothera_longirostra	Nectarivore	Forest	Moderate	Non-endemic	Least Concern	yes	none	sunbirds	no	no	yes	no
Ardea_alba	Vertivore	Wetland	Low	Non-endemic	Least Concern	no	none	large_wader	yes	yes	no	no
Ardea_cinerea	Vertivore	Wetland	Low	Non-endemic	Least Concern	no	none	large_wader	yes	no	no	no
Ardea_intermedia	Vertivore	Wetland	Low	Non-endemic	Least Concern	no	none	large_wader	yes	yes	no	no
Ardea_purpurea	Vertivore	Wetland	Low	Non-endemic	Least Concern	no	none	large_wader	yes	yes	no	no
Ardeola_grayii	Vertivore	Wetland	Low	Non-endemic	Least Concern	no	Ardeola_grayii	large_wader	yes	yes	no	no
Argya_affinis	Invertivore	NA	Low	Indian Subcontinent	Least Concern	no	none	babbler	yes	yes	yes	no
Argya_caudata	Omnivore	Shrubland	Moderate	Indian Subcontinent	Least Concern	yes	none	babbler	yes	yes	no	no
Argya_striata	Invertivore	NA	Low	Indian Subcontinent	Least Concern	no	Argya_striata	babbler	yes	yes	yes	no
Athene_brama	Invertivore	Human Modified	Low	Non-endemic	Least Concern	no	none	arboreal_invertivore	yes	yes	yes	no
Bubulcus_ibis	Omnivore	Human Modified	Low	Non-endemic	Least Concern	no	Bubulcus_ibis	large_wader	yes	yes	no	no
Cacomantis_merulinus	Invertivore	Woodland	Low	Non-endemic	Least Concern	no	none	arboreal_invertivore	yes	no	no	no
Cacomantis_passerinus	Invertivore	Woodland	Low	Indian Subcontinent	Least Concern	no	none	arboreal_invertivore	no	no	yes	no
Cacomantis_sonneratii	Invertivore	Forest	Moderate	Non-endemic	Least Concern	yes	Cacomantis_sonneratii	arboreal_invertivore	no	no	yes	no
Calandrella_dukhunensis	Omnivore	Grassland	Low	Non-endemic	Least Concern	no	none	ground_feeding_passerines	yes	yes	no	yes
Calidris_minuta	Vertivore	Grassland	High	Non-endemic	Least Concern	yes	none	small_wader_lapwing	yes	yes	no	no
Caprimulgus_asiaticus	Invertivore	Shrubland	Low	Non-endemic	Least Concern	no	none	arboreal_invertivore	no	no	yes	no
Caprimulgus_atripennis	Invertivore	Forest	Low	Indian Subcontinent	Least Concern	no	none	arboreal_invertivore	no	no	yes	no
Centropus_sinensis	Vertivore	Woodland	Low	Non-endemic	Least Concern	no	Centropus_sinensis	ground_dweller_large	yes	yes	yes	no
Ceryle_rudis	Vertivore	Wetland	Moderate	Non-endemic	Least Concern	yes	none	kingfisher	yes	yes	yes	no

Chalcophaps_indica	Omnivore	Forest	Low	Non-endemic	Least Concern	no	none	doves	no	no	yes	yes
Charadrius_dubius	Vertivore	Wetland	High	Non-endemic	Least Concern	yes	none	small_wader_lapwing	yes	no	no	no
Chloropsis_aurifrons	Omnivore	Forest	Low	Non-endemic	Least Concern	no	Chloropsis_aurifrons	arboreal_invertivore	no	no	yes	no
Chloropsis_jerdoni	Omnivore	Forest	Low	Indian Subcontinent	Least Concern	no	Chloropsis_jerdoni	leafbird	yes	yes	yes	no
Chrysocolaptes_festivus	Invertivore	Woodland	Moderate	Indian Subcontinent	Least Concern	yes	none	woodpecker	no	no	yes	no
Chrysocolaptes_guttacristatus	Invertivore	Woodland	Low	Non-endemic	Least Concern	no	none	woodpecker	yes	yes	yes	no
Chrysocolaptes_lucidus	Invertivore	Forest	Low	Non-endemic	Least Concern	no	none	woodpecker	no	no	yes	no
Cinnyris_asiaticus	Omnivore	Shrubland	Low	Non-endemic	Least Concern	no	Cinnyris_asiaticus	sunbirds	yes	yes	yes	no
Cinnyris_lotenius	Nectarivore	Forest	Low	Indian Subcontinent	Least Concern	no	none	sunbirds	no	no	yes	no
Cisticola_juncidis	Invertivore	Grassland	Low	Non-endemic	Least Concern	no	none	warblers	yes	no	no	yes
Clamator_jacobinus	Invertivore	Grassland	Low	Non-endemic	Least Concern	no	none	arboreal_invertivore	no	no	yes	no
Columba_livia	Granivore	Human Modified	Low	Non-endemic	Least Concern	no	none	doves	yes	yes	yes	no
Columba_punicea	Frugivore	Forest	High	Non-endemic	Vulnerable	yes	none	doves	no	no	yes	yes
Copsychus_malabaricus	Invertivore	Forest	Low	Non-endemic	Least Concern	no	none	flycatcher	no	no	yes	yes
Copsychus_saularis	Invertivore	Woodland	Low	Non-endemic	Least Concern	no	Copsychus_saularis	thrush	yes	yes	yes	no
Coracias_benghalensis	Omnivore	Human Modified	Moderate	Non-endemic	Least Concern	yes	Coracias_benghalensis	roller_weaver	yes	yes	no	no
Coracina_macei	Invertivore	Forest	Moderate	Non-endemic	Least Concern	yes	none	arboreal_invertivore	yes	no	yes	no
Corvus_macrorhynchos	Omnivore	Woodland	Low	Non-endemic	Least Concern	no	Corvus_macrorhynchos	crow	yes	yes	yes	no
Corvus_splendens	Omnivore	Human Modified	Low	Non-endemic	Least Concern	no	none	crow	yes	yes	yes	no
Culicicapa_ceylonensis	Invertivore	Woodland	Moderate	Non-endemic	Least Concern	yes	none	flycatcher	no	no	yes	yes
Cyornis_tickelliae	Invertivore	Forest	Low	Indian Subcontinent	Least Concern	no	Cyornis_tickelliae	flycatcher	yes	no	yes	yes
Dendrocopos_macei	Invertivore	Forest	Low	Non-endemic	Least Concern	no	none	woodpecker	no	no	yes	no
Dendronanthus_indicus	Invertivore	Forest	High	Non-endemic	Least Concern	yes	none	ground_feeding_passerines	no	no	yes	no

Dicaeum_agile	Frugivore	Forest	High	Non-endemic	Least Concern	yes	Dicaeum_agile	arboreal_frugivore	no	no	yes	no
Dicaeum_erythrorhynchos	Frugivore	Forest	Low	Indian Subcontinent	Least Concern	no	Dicaeum_erythrorhynchos	arboreal_frugivore	no	no	yes	no
Dicrurus_aeneus	Invertivore	Forest	Low	Non-endemic	Least Concern	no	Dicrurus_aeneus	arboreal_invertivore	no	no	yes	no
Dicrurus_caerulescens	Invertivore	Forest	Moderate	Indian Subcontinent	Least Concern	yes	none	arboreal_invertivore	yes	yes	yes	no
Dicrurus_leucophaeus	Invertivore	Forest	Low	Non-endemic	Least Concern	no	none	arboreal_invertivore	yes	no	yes	no
Dicrurus_macrocercus	Invertivore	Shrubland	Low	Non-endemic	Least Concern	no	Dicrurus_macrocercus	arboreal_invertivore	yes	yes	yes	no
Dicrurus_paradiseus	Invertivore	Forest	Low	Non-endemic	Least Concern	no	none	arboreal_invertivore	yes	no	yes	yes
Dinopium_benghalense	Invertivore	Woodland	Low	Indian Subcontinent	Least Concern	no	Dinopium_benghalense	woodpecker	yes	yes	yes	no
Dryocopus_javensis	Invertivore	Forest	Moderate	Non-endemic	Least Concern	yes	none	woodpecker	no	no	yes	yes
Ducula_aenea	Frugivore	Forest	Low	Non-endemic	Near Threatened	yes	none	doves	no	no	yes	yes
Dumetia_hyperythra	Invertivore	Grassland	Low	Indian Subcontinent	Least Concern	no	none	babbler	no	no	yes	no
Egretta_garzetta	Vertivore	Wetland	Low	Non-endemic	Least Concern	no	none	large_wader	yes	yes	no	no
Eremopterix_griseus	Granivore	Shrubland	Moderate	Indian Subcontinent	Least Concern	yes	none	ground_feeding_passerines	yes	yes	yes	yes
Eudynamys_scolopaceus	Frugivore	Woodland	Low	Non-endemic	Least Concern	no	none	arboreal_invertivore	yes	yes	yes	no
Euodice_malabarica	Granivore	Grassland	Low	Non-endemic	Least Concern	no	none	ground_feeding_passerines	yes	yes	no	yes
Ficedula_superciliaris	Invertivore	Forest	Low	Non-endemic	Least Concern	no	none	flycatcher	no	no	yes	yes
Galloperdix_spadicea	Omnivore	Shrubland	Low	Mainland India	Least Concern	no	none	ground_dweller_large	no	no	yes	no
Gallus_gallus	Omnivore	Forest	Low	Non-endemic	Least Concern	no	Gallus_gallus	ground_dweller_large	no	no	yes	no
Gallus_sonneratii	Omnivore	Forest	Low	Mainland India	Least Concern	no	none	ground_dweller_large	no	no	yes	no
Geokichla_citrina	Omnivore	Forest	Moderate	Non-endemic	Least Concern	yes	none	ground_feeding_passerines	no	no	yes	no
Glareola_maldivarum	Invertivore	Grassland	Low	Non-endemic	Least Concern	no	none	small_wader_lapwing	no	yes	no	no
Glaucidium_radiatum	Invertivore	Woodland	Low	Indian Subcontinent	Least Concern	no	none	arboreal_invertivore	yes	yes	yes	yes
Gracula_religiosa	Frugivore	Forest	Low	Non-endemic	Least Concern	no	none	myna	no	no	yes	yes

Gracupica_contra	Omnivore	Shrubland	Low	Non-endemic	Least Concern	no	none	myna	yes	yes	no	
Gymnoris_xanthocollis	Granivore	Forest	Low	Non-endemic	Least Concern	no	none	ground_feeding_passerines	no	no	yes	no
Halcyon_smyrnensis	Omnivore	Human Modified	Low	Non-endemic	Least Concern	no	Halcyon_smyrnensis	kingfisher	yes	yes	yes	no
Harpactes_fasciatus	Invertivore	Forest	Moderate	Indian Subcontinent	Least Concern	yes	none	arboreal_invertivore	no	no	yes	yes
Hemicircus_canente	Invertivore	Forest	Moderate	Non-endemic	Least Concern	yes	none	woodpecker	no	no	yes	yes
Hemipus_picatus	Invertivore	Shrubland	Low	Non-endemic	Least Concern	no	none	flycatcher	no	no	yes	no
Hierococcyx_varius	Invertivore	Woodland	Low	Indian Subcontinent	Least Concern	no	none	arboreal_invertivore	yes	yes	yes	no
Himalayapsitta_cyanocephala	Frugivore	Forest	Low	Indian Subcontinent	Least Concern	no	none	parakeet	no	no	yes	no
Himantopus_himantopus	Vertivore	Wetland	Low	Non-endemic	Least Concern	no	none	small_wader_lapwing	yes	no	no	no
Hydrophasianus_chirurgus	Vertivore	Wetland	Moderate	Non-endemic	Least Concern	yes	none	small_wader_lapwing	yes	no	no	no
Hypothymis_azurea	Invertivore	Forest	Low	Non-endemic	Least Concern	no	Hypothymis_azurea	flycatcher	no	no	yes	yes
Iduna_caligata	Invertivore	Shrubland	Low	Non-endemic	Least Concern	no	none	warblers	yes	yes	yes	no
Irena_puella	Frugivore	Forest	Moderate	Non-endemic	Least Concern	yes	none	arboreal_frugivore	no	no	yes	no
Ixobrychus_cinnamomeus	Vertivore	Human Modified	Low	Non-endemic	Least Concern	no	none	large_wader	yes	no	no	no
Ketupa_zeylonensis	Vertivore	Riverine	Moderate	Non-endemic	Least Concern	yes	none	arboreal_invertivore	no	no	yes	yes
Kittacincla_malabarica	Invertivore	Forest	Low	Non-endemic	Least Concern	no	Kittacincla_malabarica	ground_feeding_passerines	no	no	yes	no
Lalage_melanoptera	Invertivore	Forest	Low	Indian Subcontinent	Least Concern	no	none	arboreal_invertivore	no	no	yes	no
Lanius_vittatus	Invertivore	Shrubland	Low	Non-endemic	Least Concern	no	none	arboreal_invertivore	yes	no	no	no
Larvivora_brunnea	Invertivore	Shrubland	Moderate	Non-endemic	Least Concern	yes	none	ground_feeding_passerines	no	no	yes	yes
Leiopicus_mahrattensis	Invertivore	Woodland	High	Non-endemic	Least Concern	yes	none	woodpecker	no	no	yes	no
Leptocoma_zeylonica	Omnivore	Forest	Low	Indian Subcontinent	Least Concern	no	Leptocoma_zeylonica	sunbirds	yes	yes	yes	no
Lonchura_atricapilla	Granivore	Wetland	Low	Non-endemic	Least Concern	no	none	ground_feeding_passerines	no	yes	no	yes
Lonchura_kelaarti	Granivore	Shrubland	Low	Indian Subcontinent	Least Concern	no	none	ground_feeding_passerines	no	no	yes	yes

Lonchura_malacca	Granivore	Wetland	Low	Indian Subcontinent	Least Concern	no	none	ground_feeding_passerines	no	yes	yes	no
Lonchura_punctulata	Granivore	Grassland	Low	Non-endemic	Least Concern	no	none	ground_feeding_passerines	yes	yes	yes	yes
Lonchura_striata	Granivore	Shrubland	Low	Non-endemic	Least Concern	no	none	ground_feeding_passerines	yes	yes	yes	yes
Loriculus_vernalis	Frugivore	Forest	Low	Non-endemic	Least Concern	no	Loriculus_vernalis	parakeet	yes	no	yes	no
Machlolophus_xanthogenys	Invertivore	Forest	Low	Himalayas	Least Concern	no	Machlolophus_xanthogenys	arboreal_invertivore	yes	no	yes	no
Merops_leschenaulti	Invertivore	Woodland	Low	Non-endemic	Least Concern	no	none	arboreal_invertivore	yes	yes	yes	yes
Merops_orientalis	Invertivore	Shrubland	Low	Non-endemic	Least Concern	no	none	arboreal_invertivore	yes	yes	yes	no
Merops_philippinus	Invertivore	Wetland	Low	Non-endemic	Least Concern	no	none	arboreal_invertivore	yes	yes	yes	no
Microcarbo_niger	Vertivore	Wetland	Low	Non-endemic	Least Concern	no	none	large_wader	yes	yes	no	no
Micropternus_brachyurus	Invertivore	Forest	Low	Non-endemic	Least Concern	no	none	woodpecker	no	no	yes	no
Mirafra_affinis	Omnivore	Grassland	Low	Indian Subcontinent	Least Concern	no	none	ground_feeding_passerines	yes	yes	no	yes
Mirafra_erythroptera	Omnivore	Shrubland	Moderate	Indian Subcontinent	Least Concern	yes	none	ground_feeding_passerines	yes	yes	no	yes
Mixornis_gularis	Invertivore	Forest	Low	Non-endemic	Least Concern	no	Mixornis_gularis	babbler	yes	no	yes	yes
Monticola_cinclorhyncha	Invertivore	Forest	High	Non-endemic	Least Concern	yes	none	ground_feeding_passerines	no	no	yes	no
Monticola_solitarius	Omnivore	Rock	High	Non-endemic	Least Concern	yes	none	ground_feeding_passerines	no	no	yes	no
Motacilla_alba	Invertivore	Human Modified	Moderate	Non-endemic	Least Concern	yes	none	ground_feeding_passerines	no	no	yes	no
Motacilla_cinerea	Invertivore	Riverine	Moderate	Non-endemic	Least Concern	yes	none	ground_feeding_passerines	yes	yes	yes	no
Motacilla_flava	Invertivore	Grassland	Moderate	Non-endemic	Least Concern	yes	none	ground_feeding_passerines	no	no	yes	no
Motacilla_maderaspatensis	Invertivore	Riverine	Low	Indian Subcontinent	Least Concern	no	none	ground_feeding_passerines	yes	no	yes	no
Muscicapa_dauurica	Invertivore	Woodland	Low	Non-endemic	Least Concern	no	none	flycatcher	no	no	yes	no
Mycteria_leucocephala	Vertivore	Wetland	Low	Non-endemic	Near Threatened	yes	none	large_wader	yes	no	no	no
Ninox_scutulata	Invertivore	Forest	Low	Non-endemic	Least Concern	no	none	arboreal_invertivore	no	no	yes	yes
Nyctyornis_athertoni	Invertivore	Forest	Low	Non-endemic	Least Concern	no	none	arboreal_invertivore	no	no	yes	yes

Ocyceros_birostris	Frugivore	Woodland	Low	Indian Subcontinent	Least Concern	no	none	arboreal_frugivore	yes	no	yes	no
Oriolus_kundoo	Omnivore	Woodland	Low	Non-endemic	Least Concern	no	none	arboreal_invertivore	yes	yes	yes	no
Oriolus_xanthornus	Frugivore	Forest	Low	Non-endemic	Least Concern	no	Oriolus_xanthornus	oriole	yes	yes	yes	no
Orthotomus_sutorius	Invertivore	Shrubland	Low	Non-endemic	Least Concern	no	Orthotomus_sutorius	flycatcher	yes	yes	yes	no
Ortygornis_pondicerianus	Omnivore	NA	Low	Non-endemic	Least Concern	no	none	ground_dweller_large	yes	yes	no	no
Palaeornis_eupatria	Omnivore	Forest	Low	Non-endemic	Near Threatened	yes	none	parakeet	yes	yes	yes	no
Parus_cinereus	Invertivore	Forest	Low	Non-endemic	Least Concern	no	none	arboreal_invertivore	no	no	yes	yes
Parus_major	Invertivore	Woodland	Low	Non-endemic	Least Concern	no	Parus_major	arboreal_invertivore	no	no	yes	no
Passer_domesticus	Granivore	Human Modified	Moderate	Non-endemic	Least Concern	yes	none	ground_feeding_passerines	no	no	yes	no
Pavo_cristatus	Omnivore	Forest	Low	Indian Subcontinent	Least Concern	no	none	ground_dweller_large	no	no	yes	no
Pellorneum_ruficeps	Invertivore	Shrubland	Low	Non-endemic	Least Concern	no	Pellorneum_ruficeps	babbler	no	no	yes	yes
Pericrocotus_cinnamomeus	Invertivore	Shrubland	Low	Non-endemic	Least Concern	no	Pericrocotus_cinnamomeus	arboreal_invertivore	no	no	yes	no
Pericrocotus_flammeus	Invertivore	Forest	Moderate	Indian Subcontinent	Least Concern	yes	Pericrocotus_flammeus	arboreal_invertivore	no	no	yes	no
Phaenicophaeus_tristis	Invertivore	Forest	Low	Non-endemic	Least Concern	no	none	arboreal_invertivore	no	no	yes	yes
Phylloscopus_humei	Invertivore	Forest	Low	Non-endemic	Least Concern	no	none	warblers	no	no	yes	no
Phylloscopus_inornatus	Invertivore	Woodland	Low	Non-endemic	Least Concern	no	none	warblers	no	no	yes	no
Phylloscopus_magnirostris	Invertivore	Forest	Moderate	Non-endemic	Least Concern	yes	none	warblers	no	no	yes	no
Phylloscopus_nitidus	Invertivore	Forest	Low	Non-endemic	Least Concern	no	none	warblers	no	no	yes	no
Phylloscopus_trochiloides	Invertivore	Forest	Low	Non-endemic	Least Concern	no	Phylloscopus_trochiloides	warbler	yes	no	yes	no
Picoides_nanus	Invertivore	Woodland	Moderate	Indian Subcontinent	Least Concern	yes	none	woodpecker	no	no	yes	no
Picumnus_innominatus	Invertivore	Forest	Low	Non-endemic	Least Concern	no	none	woodpecker	no	no	yes	yes
Picus_canus	Invertivore	Forest	Low	Non-endemic	Least Concern	no	none	woodpecker	no	no	yes	no
Picus_chlorolophus	Invertivore	Forest	Low	Non-endemic	Least Concern	no	none	woodpecker	no	no	yes	no

Picus_xanthopygaeus	Invertivore	Woodland	Low	Non-endemic	Least Concern	no	none	woodpecker	no	no	yes	no
Ploceus_philippinus	Granivore	Shrubland	Low	Non-endemic	Least Concern	no	none	roller_weaver	yes	yes	no	no
Pomatorhinus_horsfieldii	Invertivore	Forest	Low	Mainland India	Least Concern	no	Pomatorhinus_horsfieldii	babbler	no	no	yes	yes
Prinia_inornata	Invertivore	Shrubland	Low	Non-endemic	Least Concern	no	none	warblers	yes	yes	no	no
Prinia_socialis	Invertivore	Grassland	Low	Indian Subcontinent	Least Concern	no	Prinia_socialis	warblers	yes	yes	yes	no
Prinia_sylvatica	Invertivore	Shrubland	Low	Indian Subcontinent	Least Concern	no	none	warblers	yes	no	yes	no
Psilopogon_haemacephalus	Frugivore	Woodland	Low	Non-endemic	Least Concern	no	Psilopogon_haemacephalus	barbet	yes	yes	yes	no
Psilopogon_lineatus	Frugivore	Forest	Low	Non-endemic	Least Concern	no	none	arboreal_frugivore	no	no	yes	no
Psilopogon_zeylanicus	Frugivore	Forest	Low	Indian Subcontinent	Least Concern	no	Psilopogon_zeylanicus	barbet	yes	yes	yes	no
Pycnonotus_cafer	Omnivore	Shrubland	Low	Non-endemic	Least Concern	no	Pycnonotus_cafer	arboreal_frugivore	yes	yes	yes	no
Pycnonotus_jocosus	Omnivore	Shrubland	Low	Non-endemic	Least Concern	no	Pycnonotus_jocosus	arboreal_frugivore	yes	yes	yes	no
Pycnonotus_luteolus	Frugivore	Shrubland	Low	Indian Subcontinent	Least Concern	no	Pycnonotus_luteolus	arboreal_frugivore	yes	no	yes	no
Pycnonotus_xantholaemus	Frugivore	Forest	Moderate	Southern Deccan Plateau	Vulnerable	yes	none	arboreal_frugivore	no	no	yes	yes
Rhipidura_albicollis	Invertivore	Forest	Low	Non-endemic	Least Concern	no	none	flycatcher	no	no	yes	no
Rhipidura_albogularis	Invertivore	Forest	Low	Mainland India	Least Concern	no	none	flycatcher	no	no	yes	no
Rhipidura_aureola	Invertivore	Forest	Moderate	Non-endemic	Least Concern	yes	none	flycatcher	no	no	yes	no
Rubigula_flaviventris	Frugivore	Forest	Low	Non-endemic	Least Concern	no	Rubigula_flaviventris	bulbul	yes	no	yes	no
Saxicola_caprata	Invertivore	Shrubland	Low	Non-endemic	Least Concern	no	none	ground_feeding_passerines	yes	yes	no	no
Saxicola_torquatus	Invertivore	Shrubland	Low	Non-endemic	Least Concern	no	none	ground_feeding_passerines	yes	yes	no	no
Saxicoloides_fulicatus	Invertivore	Shrubland	Low	Indian Subcontinent	Least Concern	no	none	ground_feeding_passerines	yes	yes	yes	no
Sitta_castanea	Invertivore	Forest	Moderate	Indian Subcontinent	Least Concern	yes	none	arboreal_invertivore	no	no	yes	yes
Sitta_frontalis	Invertivore	Forest	Moderate	Non-endemic	Least Concern	yes	Sitta_frontalis	arboreal_invertivore	no	no	yes	no
Spilopelia_chinensis	Granivore	Woodland	Low	Non-endemic	Least Concern	no	Spilopelia_chinensis	doves	yes	yes	yes	no

Spilopelia_senegalensis	Granivore	Woodland	Low	Non-endemic	Least Concern	no	none	doves	yes	yes	yes	no
Spilopelia_suratensis	Granivore	Woodland	Low	Non-endemic	Least Concern	no	Spilopelia_suratensis	medium_doves	yes	yes	yes	no
Sterna_aurantia	Vertivore	Riverine	Moderate	Non-endemic	Vulnerable	yes	none	small_wader_lapwing	yes	no	no	no
Streptopelia_decaocto	Omnivore	Human Modified	Low	Non-endemic	Least Concern	no	none	doves	yes	yes	yes	no
Streptopelia_orientalis	Omnivore	Woodland	Low	Non-endemic	Least Concern	no	none	doves	yes	no	yes	no
Streptopelia_tranquebarica	Granivore	Woodland	Low	Non-endemic	Least Concern	no	none	doves	yes	yes	no	no
Strix_leptogrammica	Vertivore	Forest	Low	Non-endemic	Least Concern	no	none	arboreal_invertivore	no	no	yes	yes
Sturnia_pagodarum	Omnivore	Forest	Low	Non-endemic	Least Concern	no	none	myna	yes	no	yes	no
Tephrodornis_pondicerianus	Invertivore	Forest	Moderate	Non-endemic	Least Concern	yes	none	arboreal_invertivore	no	no	yes	no
Tephrodornis_virgatus	Invertivore	Forest	Moderate	Non-endemic	Least Concern	yes	none	arboreal_invertivore	no	no	yes	yes
Terpsiphone_paradisi	Invertivore	Woodland	Low	Non-endemic	Least Concern	no	none	flycatcher	no	no	yes	no
Threskiornis_melanocephalus	Vertivore	Wetland	Low	Non-endemic	Near Threatened	yes	none	large_wader	no	yes	no	no
Treron_bicinctus	Frugivore	Forest	Moderate	Non-endemic	Least Concern	yes	none	doves	no	no	yes	no
Treron_phoenicopterus	Frugivore	Forest	Low	Non-endemic	Least Concern	no	none	doves	no	yes	yes	no
Tringa_ochropus	Vertivore	Wetland	Low	Non-endemic	Least Concern	no	none	small_wader_lapwing	yes	no	no	no
Turdus_simillimus	Omnivore	Forest	Low	Indian Subcontinent	Least Concern	no	none	ground_feeding_passerines	no	no	yes	no
Turdus_unicolor	Invertivore	Forest	Low	Indian Subcontinent	Least Concern	no	none	ground_feeding_passerines	no	no	yes	no
Upupa_epops	Invertivore	Grassland	Moderate	Non-endemic	Least Concern	yes	none	ground_dweller_large	yes	no	yes	no
Vanellus_indicus	Invertivore	Wetland	Low	Non-endemic	Least Concern	no	Vanellus_indicus	small_wader_lapwing	yes	yes	yes	no
Vanellus_malabaricus	Invertivore	Grassland	Low	Indian Subcontinent	Least Concern	no	none	small_wader_lapwing	yes	yes	no	yes
Zosterops_palpebrosus	Omnivore	Forest	Low	Non-endemic	Least Concern	no	Zosterops_palpebrosus	arboreal_frugivore	yes	no	yes	no

Supplementary Table 7. Species-wise relative abundance in each farming system when compared to a natural forest baseline. The posterior distribution (PD) and estimated percentage difference in abundance (with lower and upper 95% Bayesian credible intervals) are shown.

	ZBNF				Agrichemical			
**Species**	**PD**	**% difference**	**% difference lower CI**	**% difference upper CI**	**PD**	**% difference**	**% difference lower CI**	**% difference upper CI**
Acridotheres_fuscus	74.19	-79.82	-100.00	339.19	51.05	7.27	-100.00	2702.35
Acridotheres_ginginianus	70.94	215.42	-99.97	4190.42	81.36	760.74	-99.98	21014.98
Acridotheres_tristis	100.00	85141.43	7675.30	367193.87	100.00	292554.74	26839.53	1259461.21
Acrocephalus_dumetorum	96.03	228.16	-42.08	693.70	98.59	711.11	-49.23	2763.24
Actitis_hypoleucos	70.20	276.58	-99.81	11137.15	94.80	5003.06	-99.77	133954.51
Aegithina_tiphia	100.00	-99.56	-100.00	-96.49	99.44	-99.00	-100.00	-89.17
Aethopyga_siparaja	91.15	-100.00	-100.00	-34.04	82.03	-99.93	-100.00	1372.27
Alauda_gulgula	100.00	19053.81	883.54	118678.37	100.00	95533.98	5460.55	582728.64
Alcedo_atthis	82.75	757.07	-99.74	19897.20	97.98	11212.95	-97.73	269592.95
Alcedo_hercules	84.84	-87.87	-99.99	61.64	67.89	188.10	-99.97	4550.25
Alcippe_poioicephala	99.98	-99.89	-100.00	-98.14	98.68	-99.48	-100.00	-87.87
Alexandrinus_krameri	100.00	6262.27	2439.29	11008.64	100.00	13670.53	5250.11	26299.67
Amandava_amandava	66.18	204.60	-99.99	12017.65	54.75	40.48	-99.99	6081.16
Amaurornis_phoenicurus	94.21	3195.69	-99.69	68510.35	99.96	60584.15	-37.77	907426.33
Ammomanes_phoenicura	100.00	15941.23	169.56	136893.17	99.84	6583.59	-73.97	66931.32
Anastomus_oscitans	99.93	17751.18	-52.94	260774.81	100.00	104506.77	353.64	1434603.11
Anhinga_melanogaster	58.50	-41.70	-99.99	1393.95	86.01	1545.65	-99.99	45242.55
Anthracoceros_coronatus	98.35	-99.71	-100.00	-89.65	87.15	-96.34	-100.00	51.68
Anthus_godlewskii	57.53	42.35	-99.80	1710.88	98.05	1877.04	-82.86	18713.37
Anthus_rufulus	100.00	38345.04	2784.18	181944.67	100.00	106488.98	10799.89	507077.44
Arachnothera_longirostra	88.70	-99.99	-100.00	56.36	79.14	-99.81	-100.00	2983.77
Ardea_alba	99.91	20584.77	-61.38	306058.01	99.99	30950.57	-42.60	419760.57
Ardea_cinerea	82.10	834.60	-99.81	25068.01	93.61	5385.45	-99.63	185191.23
Ardea_intermedia	100.00	65802.70	978.18	789254.85	99.99	19387.80	116.53	223618.37

Ardea_purpurea	53.24	-17.35	-99.98	1556.04	83.55	737.83	-99.86	15217.64
Ardeola_grayii	100.00	44803.36	756.92	453985.29	100.00	83649.82	1659.01	850576.93
Argya_affinis	96.78	-75.93	-97.23	-33.19	50.39	0.87	-93.72	231.44
Argya_caudata	97.88	4184.06	-97.78	43542.35	99.93	62621.51	-69.98	796982.25
Argya_striata	81.91	56.60	-44.35	205.46	79.04	55.26	-59.43	214.73
Athene_brama	88.03	459.20	-94.36	3579.63	99.94	13606.43	2.24	109412.26
Bubulcus_ibis	100.00	67730.92	6130.22	312095.48	100.00	69338.38	5721.76	322711.03
Cacomantis_merulinus	67.60	176.86	-99.99	4583.08	74.90	419.62	-99.99	10800.59
Cacomantis_passerinus	98.19	-98.85	-100.00	-79.03	89.36	-95.43	-100.00	9.20
Cacomantis_sonneratii	99.84	-99.58	-100.00	-93.86	95.83	-98.20	-100.00	-64.13
Calandrella_dukhunensis	98.21	6884.21	-96.46	113719.65	99.73	45309.02	-95.46	840109.46
Calidris_minuta	93.10	3297.13	-98.83	69492.94	97.36	14944.30	-97.03	399473.33
Caprimulgus_asiaticus	93.70	-97.25	-100.00	-41.81	81.71	-90.45	-100.00	147.69
Caprimulgus_atripennis	89.95	-96.09	-100.00	1.94	76.83	-85.88	-100.00	281.28
Centropus_sinensis	62.60	18.30	-68.62	139.93	99.11	473.61	-13.35	1500.62
Ceryle_rudis	69.33	-54.03	-99.99	233.48	96.76	2450.05	-98.12	26487.61
Chalcophaps_indica	98.58	-98.46	-100.00	-79.44	84.88	-90.43	-100.00	69.52
Charadrius_dubius	53.53	26.22	-100.00	3605.61	86.69	1763.13	-99.94	54574.89
Chloropsis_aurifrons	99.90	-99.55	-100.00	-94.75	93.69	-96.85	-100.00	-44.64
Chloropsis_jerdoni	93.20	-82.04	-99.90	-20.87	99.69	2841.01	-62.62	16307.52
Chrysocolaptes_festivus	99.54	-99.53	-100.00	-92.23	94.96	-98.03	-100.00	-56.81
Chrysocolaptes_guttacristatus	97.16	-93.86	-99.99	-55.39	62.68	-43.70	-99.93	468.03
Chrysocolaptes_lucidus	91.60	-97.26	-100.00	-23.26	79.85	-89.52	-100.00	225.76
Cinnyris_asiaticus	99.85	-83.06	-96.29	-62.24	84.99	-55.98	-93.67	21.76
Cinnyris_lotenius	90.73	-99.99	-100.00	-20.79	80.78	-99.91	-100.00	1601.63
Cisticola_juncidis	64.58	124.41	-99.99	3845.90	75.25	387.70	-99.99	10208.33
Clamator_jacobinus	95.23	-97.60	-100.00	-54.03	83.38	-91.89	-100.00	119.78
Columba_livia	95.41	1544.50	-97.19	9992.10	100.00	18054.99	1763.98	51534.27

Columba_punicea	96.58	-98.46	-100.00	-67.89	75.51	-83.88	-100.00	341.30
Copsychus_malabaricus	97.69	-99.27	-100.00	-78.76	90.43	-97.10	-100.00	-5.47
Copsychus_saularis	99.35	-94.89	-100.00	-73.61	90.78	-88.21	-99.97	-8.35
Coracias_benghalensis	100.00	21445.45	1669.47	86639.19	100.00	516706.61	21511.58	2253633.81
Coracina_macei	80.89	-85.50	-100.00	144.89	51.46	8.44	-99.94	1979.63
Corvus_macrorhynchos	100.00	5727.80	1074.12	13884.04	100.00	6115.74	1399.43	14921.73
Corvus_splendens	100.00	1222.74	93.41	3471.26	99.94	1033.03	58.85	2849.69
Culicicapa_ceylonensis	99.64	-99.62	-100.00	-93.07	95.61	-98.31	-100.00	-60.79
Cyornis_tickelliae	99.21	-97.23	-100.00	-80.13	92.24	-94.48	-100.00	-26.27
Dendrocopos_macei	96.68	-98.15	-100.00	-67.11	86.36	-93.31	-100.00	57.05
Dendronanthus_indicus	86.68	-94.35	-100.00	46.64	72.29	-80.41	-100.00	511.55
Dicaeum_agile	99.99	-99.85	-100.00	-97.41	93.48	-98.07	-100.00	-43.61
Dicaeum_erythrorhynchos	100.00	-99.91	-100.00	-98.54	95.91	-98.71	-100.00	-70.96
Dicrurus_aeneus	99.83	-99.68	-100.00	-94.97	96.48	-98.63	-100.00	-71.20
Dicrurus_caerulescens	92.48	-80.40	-99.80	-16.49	81.45	-78.89	-99.98	97.67
Dicrurus_leucophaeus	61.85	-37.03	-99.96	356.88	56.04	33.04	-99.91	1429.75
Dicrurus_macrocercus	100.00	1408.88	536.93	2605.33	100.00	2427.65	797.97	4525.34
Dicrurus_paradiseus	93.63	-87.25	-99.92	-25.97	79.79	-79.42	-99.99	120.24
Dinopium_benghalense	63.00	-23.97	-93.46	120.80	99.94	4066.13	3.94	23406.78
Dryocopus_javensis	92.11	-96.23	-100.00	-23.46	78.53	-86.91	-100.00	237.96
Ducula_aenea	96.39	-98.02	-100.00	-64.80	73.24	-80.88	-100.00	441.82
Dumetia_hyperythra	95.49	-97.88	-100.00	-57.84	84.93	-92.25	-100.00	83.10
Egretta_garzetta	100.00	351143.92	4838.16	3891580.75	100.00	380952.31	2878.83	4123249.46
Eremopterix_griseus	80.45	-77.93	-99.98	95.29	85.30	-79.42	-99.92	41.83
Eudynamys_scolopaceus	99.73	841.86	-1.94	2520.94	99.18	715.58	-42.64	2629.71
Euodice_malabarica	99.15	6158.66	-96.98	73113.96	88.85	860.79	-99.51	10656.94
Ficedula_superciliaris	90.39	-96.28	-100.00	-3.60	77.50	-86.77	-100.00	286.21
Galloperdix_spadicea	90.63	-92.74	-100.00	-7.77	65.54	-60.13	-99.99	581.12

Gallus_gallus	99.78	-99.16	-100.00	-91.02	89.93	-94.66	-100.00	0.72
Gallus_sonneratii	92.86	-95.45	-100.00	-30.86	71.61	-74.93	-100.00	412.17
Geokichla_citrina	98.64	-98.38	-100.00	-80.22	84.65	-89.84	-100.00	81.48
Glareola_maldivarum	53.08	-18.00	-100.00	3135.10	69.48	-77.75	-100.00	1438.52
Glaucidium_radiatum	97.10	868.48	-75.09	4764.38	86.55	548.29	-98.32	5634.38
Gracula_religiosa	95.24	-98.98	-100.00	-66.86	77.18	-88.53	-100.00	376.03
Gracupica_contra	100.00	28254.08	1406.33	168015.75	100.00	26449.41	871.75	167171.15
Gymnoris_xanthocollis	90.19	-94.47	-100.00	-3.04	81.71	-88.55	-100.00	154.60
Halcyon_smyrnensis	96.41	-89.51	-97.65	-74.68	95.24	-85.32	-97.45	-59.67
Harpactes_fasciatus	99.53	-99.52	-100.00	-91.83	95.35	-98.02	-100.00	-57.43
Hemicircus_canente	98.26	-98.93	-100.00	-79.83	90.93	-95.71	-100.00	-9.67
Hemipus_picatus	99.41	-99.63	-100.00	-91.50	94.78	-98.36	-100.00	-57.22
Hierococcyx_varius	60.56	22.10	-83.33	218.86	70.71	92.58	-96.42	800.50
Himalayapsitta_cyanocephala	99.74	-99.75	-100.00	-95.32	91.00	-97.22	-100.00	-12.18
Himantopus_himantopus	63.99	175.20	-99.98	11065.62	95.93	8914.39	-99.72	288756.15
Hydrophasianus_chirurgus	64.88	165.11	-99.97	7996.46	90.39	2836.42	-99.95	89359.34
Hypothymis_azurea	99.94	-99.69	-100.00	-95.43	97.23	-98.68	-100.00	-73.72
Iduna_caligata	99.98	2738.27	31.47	10905.23	99.26	2945.43	-66.48	21066.88
Irena_puella	90.35	-96.07	-100.00	-4.24	63.05	-63.32	-100.00	1222.00
Ixobrychus_cinnamomeus	74.56	375.55	-99.95	11670.11	91.85	3750.43	-99.50	114332.87
Ketupa_zeylonensis	79.24	-87.28	-100.00	234.06	56.76	55.14	-100.00	4055.32
Kittacincla_malabarica	99.94	-99.72	-100.00	-95.56	97.10	-98.76	-100.00	-73.65
Lalage_melanoptera	86.31	-93.67	-100.00	53.27	71.06	-78.80	-100.00	567.95
Lanius_vittatus	64.24	120.01	-99.99	3041.80	72.09	285.78	-99.98	8034.30
Larvivora_brunnea	98.56	-98.83	-100.00	-80.56	90.58	-95.48	-100.00	-9.10
Leiopicus_mahrattensis	98.53	-98.69	-100.00	-79.18	89.40	-94.89	-100.00	7.87
Leptocoma_zeylonica	90.21	134.96	-58.06	457.00	98.90	974.44	-79.31	4855.77
Lonchura_atricapilla	68.75	361.58	-100.00	40703.53	60.34	143.25	-100.00	39175.96

Lonchura_kelaarti	62.91	-57.67	-100.00	939.56	53.90	-24.37	-100.00	2316.90
Lonchura_malacca	51.08	9.12	-100.00	3998.88	67.54	-70.47	-100.00	1564.33
Lonchura_punctulata	100.00	20176.02	22.60	128284.58	97.98	5227.49	-94.67	64185.57
Lonchura_striata	100.00	9080.29	199.03	33026.99	96.55	3839.42	-98.84	40369.57
Loriculus_vernalis	99.91	-98.96	-100.00	-92.21	77.68	-79.24	-100.00	179.61
Machlolophus_xanthogenys	98.96	-97.17	-100.00	-78.63	92.06	-94.28	-100.00	-23.65
Merops_leschenaulti	91.51	-86.61	-99.94	-11.91	91.50	-88.61	-100.00	-16.11
Merops_orientalis	82.21	228.89	-94.91	1066.97	90.01	744.45	-98.58	6117.46
Merops_philippinus	63.03	69.44	-99.82	1170.09	96.19	1534.86	-96.59	13061.43
Microcarbo_niger	87.59	767.13	-99.44	12823.09	99.95	34073.90	-55.86	543373.43
Micropternus_brachyurus	99.21	-99.31	-100.00	-88.20	93.34	-97.08	-100.00	-39.90
Mirafra_affinis	99.98	7003.76	95.85	47629.40	100.00	7191.77	58.93	51214.12
Mirafra_erythroptera	100.00	9680.46	127.14	64729.91	99.99	7131.93	52.03	53106.19
Mixornis_gularis	99.90	-99.79	-100.00	-96.45	97.88	-98.99	-100.00	-79.57
Monticola_cinclorhyncha	92.99	-96.67	-100.00	-32.36	80.25	-88.63	-100.00	189.20
Monticola_solitarius	91.68	-94.87	-100.00	-18.17	69.43	-71.21	-100.00	464.50
Motacilla_alba	92.29	-96.62	-100.00	-28.99	79.38	-88.35	-100.00	186.95
Motacilla_cinerea	99.83	-98.65	-100.00	-90.31	97.89	-96.74	-100.00	-71.39
Motacilla_flava	86.61	-94.05	-100.00	53.37	71.81	-78.55	-100.00	511.15
Motacilla_maderaspatensis	93.69	1264.06	-95.95	12159.66	92.33	1601.08	-97.50	25443.59
Muscicapa_dauurica	92.45	-96.96	-100.00	-32.63	81.43	-89.17	-100.00	167.13
Mycteria_leucocephala	74.73	374.60	-99.98	11564.74	91.98	3348.57	-99.74	101073.49
Ninox_scutulata	90.43	-95.85	-100.00	-3.66	76.86	-85.65	-100.00	274.94
Nyctyornis_athertoni	98.43	-99.26	-100.00	-83.71	91.83	-97.12	-100.00	-24.16
Ocyceros_birostris	59.20	56.50	-99.96	2272.25	80.19	672.60	-99.89	17548.17
Oriolus_kundoo	91.20	363.99	-90.86	1732.30	99.24	2958.69	-79.50	20908.64
Oriolus_xanthornus	89.41	-71.45	-98.18	5.51	99.66	3347.27	-53.54	22756.63
Orthotomus_sutorius	64.35	-12.21	-58.50	47.95	81.61	-38.18	-81.79	26.12

Ortygornis_pondicerianus	98.16	1325.92	-79.73	9229.08	99.19	6373.65	-91.00	61993.99
Palaeornis_eupatria	82.85	-81.44	-99.99	89.08	94.35	501.35	-94.11	2635.51
Parus_cinereus	98.36	-99.27	-100.00	-82.93	91.51	-96.94	-100.00	-18.95
Parus_major	99.78	-99.63	-100.00	-94.14	95.88	-98.46	-100.00	-62.92
Passer_domesticus	73.14	-78.84	-100.00	467.43	63.04	-58.51	-100.00	1198.27
Pavo_cristatus	97.79	-98.11	-100.00	-73.95	82.30	-87.65	-100.00	123.94
Pellorneum_ruficeps	99.98	-99.86	-100.00	-97.83	98.40	-99.32	-100.00	-85.71
Pericrocotus_cinnamomeus	99.98	-99.84	-100.00	-97.70	98.30	-99.26	-100.00	-84.71
Pericrocotus_flammeus	99.98	-99.83	-100.00	-97.28	98.10	-99.20	-100.00	-83.49
Phaenicophaeus_tristis	98.90	-99.11	-100.00	-84.79	92.26	-96.58	-100.00	-26.12
Phylloscopus_humei	94.60	-98.20	-100.00	-52.28	83.91	-93.24	-100.00	113.99
Phylloscopus_inornatus	95.49	-97.85	-100.00	-56.84	83.26	-91.70	-100.00	111.47
Phylloscopus_magnirostris	98.29	-99.20	-100.00	-82.47	91.76	-96.91	-100.00	-22.83
Phylloscopus_nitidus	98.16	-99.22	-100.00	-82.59	90.75	-96.97	-100.00	-10.03
Phylloscopus_trochiloides	62.19	26.83	-80.10	228.22	63.91	-41.85	-99.78	335.13
Picoides_nanus	99.09	-99.24	-100.00	-87.58	92.66	-96.98	-100.00	-35.19
Picumnus_innominatus	99.51	-99.28	-100.00	-89.88	93.40	-97.02	-100.00	-43.15
Picus_canus	86.39	-94.42	-100.00	60.28	72.41	-81.36	-100.00	524.41
Picus_chlorolophus	99.60	-99.40	-100.00	-90.64	94.09	-97.64	-100.00	-50.06
Picus_xanthopygaeus	90.19	-96.02	-100.00	-2.34	76.94	-86.29	-100.00	285.03
Ploceus_philippinus	97.96	23141.81	-92.07	449738.69	100.00	156615.54	1366.63	1358566.26
Pomatorhinus_horsfieldii	99.99	-99.86	-100.00	-97.89	98.48	-99.32	-100.00	-86.69
Prinia_inornata	100.00	17203.43	533.31	119187.86	100.00	20619.42	670.16	137508.97
Prinia_socialis	99.89	796.11	44.96	2433.44	100.00	3624.16	367.94	9871.84
Prinia_sylvatica	93.65	462.12	-88.73	2312.80	65.21	108.35	-99.88	2065.96
Psilopogon_haemacephalus	99.68	-83.25	-96.93	-58.91	82.66	317.03	-94.97	2926.01
Psilopogon_lineatus	79.81	-89.00	-100.00	211.68	50.36	-2.80	-100.00	3306.31
Psilopogon_zeylanicus	99.86	-96.04	-99.95	-83.11	87.95	-88.14	-99.99	24.83

Pycnonotus_cafer	51.36	1.25	-47.50	56.80	95.48	124.37	-30.33	335.95
Pycnonotus_jocosus	100.00	-98.98	-99.88	-97.49	100.00	-96.47	-99.43	-90.89
Pycnonotus_luteolus	97.86	-91.41	-99.86	-58.93	67.41	-59.71	-99.99	446.72
Pycnonotus_xantholaemus	93.50	-97.48	-100.00	-35.88	69.36	-75.07	-100.00	776.73
Rhipidura_albicollis	92.08	-96.17	-100.00	-24.78	78.60	-86.37	-100.00	227.16
Rhipidura_albogularis	85.55	-93.52	-100.00	64.66	70.31	-77.80	-100.00	544.59
Rhipidura_aureola	98.88	-99.30	-100.00	-86.01	92.58	-97.17	-100.00	-31.38
Rubigula_flaviventris	95.26	-90.40	-99.95	-41.89	64.20	-53.28	-99.99	563.98
Saxicola_caprata	74.53	-78.51	-100.00	337.64	52.44	-12.61	-99.97	1374.57
Saxicola_torquatus	54.14	-16.58	-99.87	630.64	98.49	3543.91	-91.33	35485.31
Saxicoloides_fulicatus	90.98	-84.85	-99.99	-6.64	56.75	-24.63	-99.65	513.81
Sitta_castanea	99.19	-99.52	-100.00	-90.08	94.38	-98.02	-100.00	-51.35
Sitta_frontalis	99.96	-99.76	-100.00	-96.23	97.45	-98.92	-100.00	-76.79
Spilopelia_chinensis	54.34	-4.93	-63.96	76.31	93.53	165.34	-49.37	550.69
Spilopelia_senegalensis	100.00	15957.00	2169.06	45681.77	100.00	11942.22	606.20	40390.42
Spilopelia_suratensis	54.18	-6.01	-80.15	106.48	100.00	2431.07	393.68	5955.38
Sterna_aurantia	79.95	1425.07	-99.98	111990.99	97.80	30512.39	-99.97	1388534.71
Streptopelia_decaocto	56.59	-14.59	-95.34	193.52	96.68	1388.59	-94.05	8958.84
Streptopelia_orientalis	60.11	-34.90	-99.94	412.27	61.25	83.30	-99.98	2410.36
Streptopelia_tranquebarica	95.59	3372.92	-97.35	51782.12	96.60	6458.06	-99.06	119206.26
Strix_leptogrammica	93.98	-96.88	-100.00	-43.44	62.71	-57.58	-100.00	865.34
Sturnia_pagodarum	74.58	-76.70	-100.00	304.96	66.45	156.01	-99.97	4631.74
Tephrodornis_pondicerianus	99.64	-99.59	-100.00	-92.83	95.35	-98.25	-100.00	-58.95
Tephrodornis_virgatus	99.85	-99.75	-100.00	-95.67	97.20	-98.94	-100.00	-76.47
Terpsiphone_paradisi	93.00	-96.93	-100.00	-31.15	80.14	-88.67	-100.00	187.72
Threskiornis_melanocephalus	96.71	9970.15	-96.97	271164.13	100.00	79171.70	49.87	1262692.50
Treron_bicinctus	92.45	-96.69	-100.00	-27.15	65.98	-67.46	-100.00	897.23
Treron_phoenicopterus	87.21	-90.66	-100.00	36.60	59.54	68.43	-99.99	2483.83

Tringa_ochropus	68.55	315.90	-99.99	18716.08	95.79	9856.89	-99.60	324273.89
Turdus_simillimus	96.83	-97.85	-100.00	-67.75	80.68	-86.84	-100.00	148.00
Turdus_unicolor	85.30	-93.97	-100.00	78.79	70.80	-78.07	-100.00	592.77
Upupa_epops	58.94	29.11	-98.11	431.41	61.86	63.80	-99.86	1327.47
Vanellus_indicus	97.65	439.70	-44.81	1911.93	100.00	1883.59	126.82	6807.14
Vanellus_malabaricus	98.98	5212.97	-98.89	38931.37	99.49	19608.18	-98.59	215150.16
Zosterops_palpebrosus	99.99	-99.64	-100.00	-95.71	94.73	-97.37	-100.00	-52.65

Supplementary Table 8. Effects of the transition to ZBNF, and of farming system-wise effects of increasing landscape-level agricultural productivity and of economic profit for each species. The posterior distribution (PD) and estimated percentage change in abundance (with lower and upper 95% Bayesian credible intervals) per one unit increase in yield/profit are shown. The species ID numbers corresponds to those depicted in Figure 2.b.

		Effect of the transition to ZBNF				Effect of agricultural productivity in ZBNF systems				Effect of agricultural productivity in agrichemical systems				Effect of economic profit in ZBNF systems				Effect of economic profit in agrichemical systems			
**Species**	**ID number**	PD	% change	% change lower CI	% change upper CI	PD	% change	% change lower CI	% change upper CI	PD	% change	% change lower CI	% change upper CI	PD	% change	% change lower CI	% change upper CI	PD	% change	% change lower CI	% change upper CI
Acridotheres_ginginianus	**44**	57.95	18.11	-90.12	212.09	66.58	-36.70	-98.86	148.19	86.99	-83.64	-99.99	27.75	88.91	-32.90	-68.34	8.02	77.99	-49.06	-97.82	59.61
Acridotheres_tristis	**58**	99.25	40.26	10.58	72.38	79.28	10.74	-12.43	33.92	100.00	-68.63	-77.64	-58.51	65.66	4.01	-11.52	20.82	100.00	-40.82	-51.94	-29.80
Acrocephalus_dumetorum	63	85.84	46.13	-32.30	135.95	99.21	-67.94	-89.70	-39.80	99.96	-90.71	-99.22	-74.41	54.01	1.92	-34.81	38.69	98.23	-54.04	-82.74	-21.72
Actitis_hypoleucos	**88**	86.91	119.28	-68.16	469.07	96.43	410.27	-73.32	1581.21	62.78	-32.33	-99.31	240.49	80.63	-23.03	-59.77	20.77	79.73	-49.67	-96.74	49.93
Aegithina_tiphia	**50**	63.93	29.00	-82.07	210.51	53.81	-9.21	-95.27	210.48	81.69	-71.28	-99.84	58.61	83.45	-26.58	-64.90	16.52	92.33	-69.21	-97.83	-9.40
Alauda_gulgula	**14**	94.99	-27.02	-50.01	-4.08	98.36	55.94	3.07	108.10	94.69	-22.15	-41.71	-1.93	52.71	-1.06	-26.02	26.72	100.00	-29.93	-39.47	-20.04
Alcedo_atthis	**94**	88.49	124.15	-63.42	475.65	92.24	213.85	-74.62	878.39	79.45	-63.02	-99.83	70.16	87.50	-29.22	-63.47	9.55	74.75	-36.74	-93.10	52.61
Alcedo_hercules	95	86.51	125.81	-77.17	505.74	94.80	315.24	-67.42	1266.46	85.25	-76.94	-99.97	36.25	65.84	-12.05	-55.60	41.73	89.89	-66.19	-98.41	1.15

Alexandrinus_krameri	**52**	97.41	30.15	4.70	61.14	75.48	-9.94	-32.72	14.37	100.00	-64.97	-77.89	-50.01	70.21	4.99	-11.11	21.98	62.94	5.71	-23.36	33.00
Amandava_amandava	**12**	69.11	-29.74	-92.55	74.96	55.14	16.47	-98.52	400.47	90.35	254.87	-87.79	1038.17	97.66	-58.80	-86.13	-25.86	91.44	84.56	-33.40	224.59
Amaurornis_phoenicurus	25	55.93	-8.15	-84.15	99.02	82.64	135.10	-89.96	955.65	85.33	-61.05	-98.42	28.22	91.35	-31.48	-66.17	1.56	94.89	-62.14	-94.05	-18.51
Ammomanes_phoenicura	**16**	75.66	-23.69	-65.83	32.35	74.91	-21.86	-64.01	30.66	86.55	109.25	-65.00	401.39	72.33	-12.64	-48.03	22.32	80.21	-45.69	-89.86	39.84
Anastomus_oscitans	11	79.51	-32.74	-76.47	27.28	79.20	49.16	-47.26	191.61	68.98	-15.67	-59.55	37.79	96.24	-33.69	-58.35	-6.97	99.13	-30.73	-51.04	-10.92
Anhinga_melanogaster	**98**	89.30	139.85	-64.61	529.08	99.11	623.70	-28.79	2215.10	70.19	-53.20	-99.88	157.44	77.30	-20.71	-57.72	26.62	85.16	-58.47	-97.33	21.48
Anthus_godlewskii	**114**	100.00	353.86	120.14	656.31	100.00	1023.35	241.92	2188.51	97.71	148.88	5.32	341.71	93.86	42.26	-10.94	107.53	78.78	11.24	-12.88	33.79
Anthus_rufulus	27	67.06	-6.06	-28.00	17.69	100.00	67.07	31.40	105.54	64.90	-4.72	-22.35	16.16	80.44	10.81	-10.26	32.27	99.99	-19.23	-28.03	-10.78
Ardea_alba	**102**	97.49	161.82	-15.40	463.22	81.64	52.06	-48.70	195.03	69.68	-22.46	-80.26	45.33	84.29	-18.62	-47.61	12.39	83.31	-36.16	-80.63	20.80
Ardea_cinerea	89	85.83	119.91	-77.28	483.12	67.13	56.35	-95.08	470.99	82.99	-77.57	-99.94	63.10	86.95	-31.81	-69.48	10.49	80.46	-52.14	-97.00	41.39
Ardea_intermedia	29	60.26	-5.77	-39.93	29.09	98.23	51.56	4.00	106.70	100.00	144.44	72.25	227.39	98.89	-25.55	-40.92	-9.58	99.95	60.41	25.94	98.65
Ardea_purpurea	**110**	97.48	230.83	-34.42	677.27	100.00	1045.92	133.77	2739.67	80.93	101.97	-83.88	434.40	74.56	-15.98	-50.10	25.04	72.71	-29.77	-87.04	50.88
Ardeola_grayii	**38**	51.88	1.23	-35.51	45.67	95.91	59.68	-2.69	133.53	90.56	33.80	-11.64	83.06	95.14	-26.33	-48.82	-1.91	91.93	-24.66	-48.57	0.49
Argya_affinis	57	82.29	35.49	-28.33	113.48	58.29	-13.32	-82.24	97.14	97.20	-82.46	-98.95	-45.04	61.73	5.62	-25.35	37.37	83.00	-30.68	-67.59	19.55
Argya_caudata	**6**	93.89	-48.47	-81.74	-6.44	52.40	6.39	-94.60	256.79	84.05	-86.55	-99.96	79.88	54.31	-2.95	-50.14	45.95	90.89	-65.11	-97.40	-3.68
Argya_striata	**40**	54.88	2.72	-33.24	40.26	99.83	-59.20	-80.20	-37.99	99.88	-69.37	-87.02	-46.15	68.05	14.34	-43.97	59.88	97.28	-53.35	-79.81	-20.16
Athene_brama	23	58.76	-11.60	-74.68	89.37	54.24	-8.02	-88.94	132.30	99.39	-93.71	-99.98	-72.72	63.59	-8.85	-49.88	37.73	96.79	-69.37	-96.26	-28.90
Bubulcus_ibis	41	74.95	10.31	-14.65	37.50	55.19	1.69	-23.63	27.67	71.21	-9.07	-34.14	17.80	99.03	-24.64	-39.39	-8.39	96.86	25.25	1.02	49.48
Cacomantis_merulinus	77	80.84	84.62	-70.12	367.16	65.19	-32.61	-98.26	146.16	84.74	-78.25	-99.99	44.46	78.99	-22.61	-63.90	21.85	71.70	-37.61	-96.77	72.22

Calandrella_dukhunensis	35	50.25	0.63	-84.73	154.86	58.46	-19.96	-97.74	241.58	83.00	-81.55	-99.99	97.21	52.86	2.31	-50.69	58.81	73.95	-45.29	-96.54	97.21
Calidris_minuta	**46**	63.91	25.10	-77.49	191.56	87.75	226.59	-86.04	1243.55	55.99	19.82	-99.38	694.13	87.59	-29.51	-64.20	10.32	79.00	-46.38	-96.43	57.68
Centropus_sinensis	**30**	56.61	-4.58	-44.95	39.73	84.94	-27.43	-61.61	12.85	99.99	-86.24	-97.12	-70.74	59.59	4.09	-24.75	33.44	99.98	-75.20	-93.09	-54.05
Ceryle_rudis	67	77.63	63.75	-67.05	282.29	92.08	171.92	-59.19	627.47	97.90	-91.70	-99.96	-54.50	79.55	-23.16	-61.27	22.61	98.91	-82.82	-99.00	-50.85
Charadrius_dubius	87	85.64	118.66	-71.28	484.49	91.64	274.10	-78.91	1296.96	76.65	-66.61	-99.99	125.41	87.40	-32.57	-69.94	8.96	81.91	-55.81	-97.94	34.82
Chloropsis_jerdoni	3	96.94	-64.68	-91.18	-27.52	55.18	-8.63	-88.93	133.02	100.00	-98.46	-99.99	-93.16	88.08	36.55	-18.52	105.35	100.00	-92.53	-99.16	-81.03
Chrysocolaptes_guttacristatus	**26**	55.20	-7.45	-79.13	101.85	67.09	46.76	-89.33	331.68	92.35	-83.54	-99.92	-15.66	78.69	-20.59	-60.32	20.86	89.18	-59.37	-96.80	4.09
Cinnyris_asiaticus	5	97.65	-49.15	-74.29	-18.21	92.14	-44.03	-77.36	0.42	99.81	-76.06	-94.74	-50.33	58.98	4.67	-29.93	42.00	99.90	-70.30	-89.45	-44.10
Cisticola_juncidis	73	80.50	82.48	-73.60	363.65	61.09	-24.13	-97.33	190.99	83.53	-76.91	-99.97	58.79	80.63	-24.14	-65.67	20.37	70.78	-35.33	-97.15	81.16
Columba_livia	**4**	99.54	-61.33	-82.80	-34.69	80.49	-57.48	-96.85	58.67	100.00	-88.73	-96.21	-79.27	91.91	-31.62	-63.41	3.62	100.00	-72.13	-86.29	-57.44
Copsychus_saularis	21	62.69	-16.36	-80.23	72.75	69.64	-34.30	-93.95	93.59	90.74	-77.59	-99.89	-3.89	80.46	-20.85	-60.87	18.26	92.94	-64.49	-96.14	-10.62
Coracias_benghalensis	53	89.90	32.47	-11.31	83.13	97.40	-37.28	-60.85	-11.03	100.00	-97.38	-99.51	-94.35	65.91	-6.10	-29.26	18.01	100.00	-70.92	-84.16	-55.73
Coracina_macei	**78**	80.16	86.04	-77.76	397.27	78.56	118.31	-93.73	733.37	78.03	-68.25	-99.97	104.68	52.25	1.82	-50.18	57.06	76.21	-46.54	-97.57	65.11
Corvus_macrorhynchos	**24**	68.39	-8.28	-33.60	18.62	54.65	2.51	-29.77	39.15	66.21	7.73	-24.32	38.91	98.53	-28.81	-48.70	-8.78	72.36	5.85	-10.50	22.02
Corvus_splendens	**18**	75.24	-17.36	-52.51	23.57	83.29	-26.73	-64.72	14.36	59.49	-7.59	-50.97	46.36	96.94	-31.54	-56.03	-6.91	75.54	20.41	-23.62	73.22
Cyornis_tickelliae	**72**	80.34	82.02	-74.90	370.33	86.84	-67.60	-99.11	15.80	96.56	-94.18	-100.00	-54.87	84.41	-27.27	-67.00	15.35	88.64	-63.65	-98.22	6.01
Dicrurus_caerulescens	61	70.91	42.78	-72.21	219.17	81.44	-51.00	-97.09	37.14	85.50	-74.64	-99.94	26.59	61.44	-7.24	-50.72	38.94	85.99	-58.17	-97.34	17.17
Dicrurus_leucophaeus	**82**	83.30	102.16	-76.26	420.39	69.66	-38.30	-96.43	99.93	91.76	-84.99	-99.99	-11.18	81.30	-23.86	-64.23	17.79	78.61	-45.11	-95.59	39.63
Dicrurus_macrocercus	**48**	89.86	25.98	-7.85	66.80	56.63	3.09	-26.15	34.71	98.93	-39.46	-59.54	-17.37	80.21	-10.64	-28.82	10.17	88.41	-13.58	-30.45	3.65

Dicrurus_paradiseus	69	75.65	73.81	-78.73	391.99	88.41	-67.31	-99.00	8.59	96.00	-92.97	-100.00	-49.02	64.26	-9.98	-51.00	39.13	88.69	-63.56	-97.67	6.34
Dinopium_benghalense	33	50.25	-0.26	-64.92	87.54	88.69	-47.07	-85.45	8.14	100.00	-98.81	-99.99	-94.95	51.59	-1.01	-47.13	47.65	100.00	-90.96	-98.93	-78.92
Egretta_garzetta	**106**	100.00	191.40	56.66	364.33	98.56	-32.26	-52.58	-12.70	99.03	-62.28	-89.84	-30.75	58.66	-2.83	-22.67	18.16	98.04	-25.28	-46.80	-6.30
Eremopterix_griseus	9	76.83	-35.96	-83.39	43.10	90.09	198.29	-77.71	852.13	99.83	475.62	26.38	1223.10	99.28	-58.46	-82.00	-31.35	99.81	178.85	23.02	366.19
Eudynamys_scolopaceus	83	96.63	104.09	-11.40	256.78	83.55	-32.72	-75.71	16.01	94.95	-57.51	-92.21	-13.36	70.14	-16.32	-64.06	35.60	98.84	-62.38	-89.44	-28.18
Euodice_malabarica	1	100.00	-77.79	-90.95	-62.09	65.85	21.17	-56.77	136.31	100.00	1974.91	223.38	5521.13	56.01	-5.95	-66.00	67.07	100.00	-98.30	-99.78	-95.87
Glareola_maldivarum	**34**	50.10	0.20	-85.51	145.78	80.48	155.29	-92.78	963.44	97.23	887.48	-82.20	3089.25	97.01	-52.85	-81.72	-16.49	99.60	226.84	61.17	455.50
Glaucidium_radiatum	**100**	90.60	143.79	-56.81	517.87	97.10	-79.71	-99.11	-39.33	96.58	-89.67	-99.95	-46.15	51.91	1.18	-45.93	54.15	66.49	-25.23	-91.96	96.75
Gracupica_contra	17	81.41	-21.15	-51.17	17.87	83.93	-17.83	-44.16	9.66	53.13	-2.09	-37.21	39.98	91.53	-16.61	-33.84	2.25	67.09	-3.75	-17.92	9.83
Halcyon_smyrnensis	**66**	93.36	60.18	-13.62	152.63	58.55	-5.84	-45.54	38.53	82.98	-31.43	-72.78	18.94	84.84	-17.23	-40.62	9.79	61.59	4.72	-20.58	30.72
Hierococcyx_varius	91	96.19	121.35	-18.50	313.15	81.38	-33.33	-78.73	22.82	96.33	-76.43	-98.56	-30.87	93.44	-29.98	-59.28	1.61	87.54	-39.80	-82.94	8.85
Himantopus_himantopus	99	86.16	141.44	-72.71	592.10	98.49	901.97	-64.83	3704.63	63.64	-38.42	-99.80	300.39	59.18	-6.42	-50.83	50.37	74.24	-46.16	-97.60	86.54
Hydrophasianus_chirurgus	93	85.96	122.69	-71.09	494.40	84.63	180.26	-86.41	1012.11	78.55	-69.49	-99.88	101.63	77.04	-20.73	-58.69	26.81	86.05	-61.98	-98.65	18.93
Iduna_caligata	97	95.16	137.93	-25.43	391.08	98.60	-74.06	-95.75	-40.86	98.66	-88.62	-99.90	-56.74	65.24	-8.27	-42.25	30.03	81.98	-36.05	-86.40	20.85
Ixobrychus_cinnamomeus	85	84.89	116.87	-76.57	491.29	72.06	85.28	-93.69	699.22	82.35	-76.95	-99.98	69.73	90.89	-37.57	-71.57	3.47	81.50	-53.34	-97.80	39.98
Lanius_vittatus	**74**	79.58	82.63	-72.95	374.55	64.21	-32.00	-98.79	162.50	84.24	-78.07	-99.97	53.32	83.76	-27.20	-67.29	17.62	69.86	-36.54	-96.61	84.30
Leptocoma_zeylonica	55	80.30	34.03	-36.63	114.08	100.00	-86.51	-96.72	-71.78	100.00	-98.46	-99.97	-94.17	81.81	19.56	-19.92	65.18	99.95	-75.33	-92.50	-53.13
Lonchura_atricapilla	19	60.60	-17.08	-89.62	116.92	50.53	1.51	-98.78	363.84	75.05	148.68	-97.30	913.82	95.01	-57.49	-88.26	-15.66	75.05	59.81	-70.43	192.47

Lonchura_malacca	**8**	74.08	-37.92	-91.81	58.88	55.90	20.40	-96.84	411.08	93.40	471.56	-91.87	1748.52	97.00	-59.10	-87.73	-24.37	95.08	142.01	-26.97	316.15
Lonchura_punctulata	101	97.86	158.93	-12.20	407.83	99.61	-91.22	-99.54	-73.30	93.36	-88.53	-99.99	-27.20	89.96	-30.98	-64.20	6.55	59.04	-19.50	-94.34	209.58
Lonchura_striata	65	77.81	53.23	-63.84	255.97	99.25	-92.20	-99.55	-72.40	92.63	-93.57	-99.99	-33.43	67.81	13.44	-39.08	76.74	84.33	-65.39	-99.01	37.57
Loriculus_vernalis	111	94.89	231.33	-57.92	832.29	70.59	64.53	-86.91	423.17	97.80	-96.34	-100.00	-67.33	92.99	64.80	-13.87	162.82	99.50	-93.72	-99.90	-74.48
Machlolophus_xanthogenys	75	79.15	83.89	-75.62	377.41	86.76	-68.76	-99.27	18.40	97.01	-94.83	-100.00	-57.78	76.26	-20.39	-60.11	28.64	89.29	-64.63	-98.46	3.62
Merops_leschenaulti	**36**	50.45	0.81	-82.22	123.84	59.31	-18.98	-94.94	157.18	56.83	-15.57	-97.57	197.90	51.18	-0.81	-47.96	50.42	89.23	-60.10	-96.52	3.88
Merops_orientalis	**108**	99.86	214.77	46.61	437.86	77.13	-28.74	-74.80	32.55	98.03	-88.78	-99.91	-52.48	91.36	30.74	-11.44	76.17	92.79	-58.17	-93.44	-5.98
Merops_philippinus	**20**	62.18	-16.65	-81.63	85.99	68.24	-36.65	-98.72	138.11	96.24	-91.21	-99.95	-51.20	55.05	-3.28	-47.72	42.15	81.09	-43.72	-88.67	38.23
Microcarbo_niger	105	97.14	191.14	-30.62	523.37	99.26	344.30	-6.10	922.85	95.76	-82.77	-99.87	-32.22	65.11	-9.98	-46.19	39.75	92.53	-61.63	-95.84	-8.56
Mirafra_affinis	**60**	85.88	41.94	-26.76	129.68	86.48	43.84	-25.50	123.94	83.34	34.27	-28.02	106.30	85.49	-17.43	-42.48	10.91	84.94	13.02	-8.28	34.92
Mirafra_erythroptera	**32**	51.79	-1.54	-51.85	65.10	55.91	-4.23	-48.59	44.26	63.86	12.03	-40.61	78.44	89.86	-19.36	-42.09	4.27	50.19	-0.07	-26.76	28.75
Mixornis_gularis	**42**	58.18	14.02	-77.62	169.13	67.11	-38.97	-99.64	162.09	94.80	-93.62	-100.00	-42.19	84.46	-30.69	-73.62	16.36	90.54	-70.50	-99.12	-2.58
Motacilla_cinerea	**2**	100.00	-73.36	-83.51	-59.51	100.00	1288.29	260.70	3105.28	100.00	1142.70	212.60	2660.77	92.14	32.46	-17.96	124.72	99.03	85.93	9.94	165.03
Motacilla_maderaspatensis	**84**	84.55	110.31	-73.65	455.70	79.69	-57.07	-98.94	65.06	89.78	-83.88	-99.97	3.10	58.08	6.03	-47.02	69.50	67.64	-31.25	-95.15	107.01
Mycteria_leucocephala	**86**	84.18	118.35	-73.63	505.96	73.28	87.03	-95.60	680.30	82.28	-76.12	-99.96	77.22	90.06	-36.91	-72.76	4.02	81.61	-54.61	-97.48	39.72
Ocyceros_birostris	107	91.86	201.45	-61.43	767.13	67.83	-37.30	-99.01	140.39	96.58	-95.86	-100.00	-59.96	66.85	19.91	-57.33	111.49	97.00	-88.33	-99.74	-48.06
Oriolus_kundoo	**22**	61.75	-13.54	-74.85	70.31	91.73	-62.15	-95.42	-5.16	99.73	-94.91	-99.99	-76.51	72.21	-13.85	-49.66	25.91	96.19	-65.39	-94.78	-20.30
Oriolus_xanthornus	103	99.24	174.16	3.50	411.10	82.18	61.68	-62.92	271.61	100.00	-99.04	-100.00	-95.33	98.88	165.75	18.09	356.87	100.00	-95.47	-99.64	-86.71
Orthotomus_sutorius	**68**	99.30	70.46	9.60	131.64	96.09	-33.12	-56.56	-6.41	99.43	-54.77	-78.02	-27.97	72.59	9.36	-15.97	36.20	96.69	-27.88	-50.65	-3.89
Ortygornis_pondicerianus	**80**	86.80	98.83	-49.65	346.86	78.08	60.15	-61.50	243.87	84.71	-61.70	-98.38	33.16	91.30	-30.03	-62.61	3.51	73.40	-34.34	-94.72	93.29
Palaeornis_eupatria	43	62.38	15.38	-56.05	110.65	73.64	66.49	-81.36	375.14	97.99	-92.36	-99.96	-62.75	89.23	39.56	-24.69	111.47	96.54	-68.34	-94.74	-29.48

Phylloscopus_trochiloides	113	99.91	350.66	42.10	845.00	99.93	-83.60	-96.91	-63.63	98.76	-94.04	-99.98	-67.11	59.35	5.73	-31.68	49.90	88.50	-49.89	-92.72	7.01
Ploceus_philippinus	**10**	85.55	-35.07	-74.14	14.46	50.19	0.40	-91.54	323.89	96.76	-67.80	-90.03	-28.65	90.91	-35.69	-71.93	3.06	54.53	-12.17	-94.60	235.66
Prinia_inornata	13	83.19	-27.09	-62.71	14.63	65.51	-11.16	-51.04	37.23	63.10	7.98	-27.34	51.34	81.51	-15.82	-45.71	13.74	80.74	-8.06	-22.58	7.41
Prinia_socialis	**62**	92.43	44.40	-9.58	110.48	99.84	110.88	28.96	197.64	57.11	4.14	-31.07	44.95	78.61	12.65	-13.72	44.28	96.44	-13.44	-25.51	-1.31
Prinia_sylvatica	79	86.51	98.41	-65.79	367.60	99.79	-91.65	-99.77	-73.48	98.39	-95.72	-100.00	-69.21	59.10	6.48	-41.37	59.07	81.29	-46.40	-94.98	29.28
Psilopogon_haemacephalus	**54**	75.90	32.87	-44.55	132.71	56.73	-7.01	-64.23	64.54	99.99	-98.48	-100.00	-91.24	99.76	100.64	31.46	183.38	99.99	-96.68	-99.79	-89.01
Psilopogon_zeylanicus	**104**	93.45	179.95	-48.15	612.81	68.06	-28.75	-93.26	73.33	95.98	-92.24	-99.99	-47.95	79.88	31.34	-32.90	102.64	99.51	-92.39	-99.74	-72.36
Pycnonotus_cafer	37	52.24	0.86	-24.92	29.48	99.96	-46.07	-63.29	-27.77	100.00	-84.99	-93.06	-75.07	98.04	30.79	3.92	58.30	100.00	-65.99	-80.01	-50.30
Pycnonotus_jocosus	31	53.84	-3.82	-60.04	65.84	99.75	174.70	29.57	369.07	55.29	-5.38	-62.11	63.37	60.91	-5.13	-36.30	27.57	97.91	-49.93	-79.18	-16.55
Pycnonotus_luteolus	**112**	97.05	292.17	-58.17	930.15	90.34	-70.22	-99.10	-1.15	99.35	-98.75	-100.00	-87.54	89.96	54.96	-22.80	144.59	99.46	-93.40	-99.88	-73.55
Rubigula_flaviventris	109	93.59	220.56	-65.21	826.36	89.16	-68.81	-99.05	5.00	99.50	-98.73	-100.00	-87.19	82.88	39.78	-36.06	124.89	98.91	-91.91	-99.93	-67.37
Saxicola_caprata	**64**	73.99	47.56	-73.79	234.53	99.75	793.74	15.35	2663.12	97.13	311.61	-38.06	1003.73	58.01	-4.72	-42.55	39.94	93.13	48.52	-9.69	118.93
Saxicola_torquatus	**70**	84.50	75.38	-55.28	272.61	99.39	331.75	-0.20	863.92	91.56	-74.30	-99.55	-7.03	73.83	-13.99	-46.58	23.00	83.06	-40.81	-88.82	22.92
Saxicoloides_fulicatus	**76**	82.93	83.98	-70.18	343.16	89.89	145.38	-66.04	513.01	71.70	-45.42	-99.53	100.78	58.53	-5.26	-44.83	40.42	85.45	-51.72	-95.10	16.32
Spilopelia_chinensis	71	99.34	79.50	14.41	149.03	63.95	-9.60	-47.46	35.50	99.86	-79.43	-94.82	-56.84	80.41	-11.08	-31.91	8.80	99.79	-68.32	-87.60	-43.11
Spilopelia_senegalensis	**56**	87.55	34.88	-18.22	93.69	100.00	-79.03	-89.87	-66.68	99.95	-81.91	-96.30	-62.10	99.35	-35.09	-55.25	-13.31	89.76	-52.82	-87.05	4.11
Spilopelia_suratensis	7	98.40	-45.67	-69.53	-19.49	56.65	6.81	-53.45	88.41	100.00	-90.37	-97.37	-80.95	72.04	-12.81	-42.12	23.26	100.00	-85.48	-95.20	-72.22
Sterna_aurantia	81	80.95	100.35	-78.06	521.01	96.09	820.28	-80.45	3953.97	52.31	-9.05	-99.83	801.64	54.98	3.52	-45.81	68.15	67.39	-36.69	-98.24	139.81
Streptopelia_decaocto	39	51.73	2.66	-69.75	109.52	82.70	74.30	-59.24	292.23	94.16	-86.71	-99.81	-37.34	77.61	13.72	-18.48	47.30	93.95	-60.55	-92.04	-13.77

Streptopelia_orientalis	47	62.84	25.94	-87.14	219.88	81.53	-59.60	-98.93	45.50	95.01	-92.03	-100.00	-41.52	73.70	-17.60	-59.72	28.32	86.96	-61.32	-98.11	15.54
Streptopelia_tranquebarica	15	69.40	-26.77	-84.31	63.84	68.48	-43.14	-98.17	153.31	67.90	-59.50	-99.96	488.09	94.15	-38.68	-70.84	-4.18	78.36	-52.23	-98.13	65.78
Sturnia_pagodarum	51	63.20	29.03	-82.45	242.16	72.55	84.94	-94.72	570.32	85.91	-79.07	-99.87	35.79	54.99	3.76	-47.26	59.30	85.98	-60.48	-98.07	18.29
Threskiornis_melanocephalus	45	59.63	19.32	-89.84	197.41	90.68	332.01	-89.67	1771.33	70.09	21.10	-45.76	103.50	69.99	-13.81	-55.23	34.06	69.41	-19.22	-69.18	42.20
Treron_phoenicopterus	**92**	84.65	121.90	-74.33	510.31	69.29	-42.94	-99.45	152.07	99.14	-98.65	-100.00	-85.31	88.03	58.92	-32.49	170.69	99.68	-94.96	-99.92	-79.22
Tringa_ochropus	**96**	85.20	127.02	-75.83	525.72	97.48	641.11	-78.03	2744.33	64.29	-41.99	-99.98	326.02	62.90	-8.82	-51.54	44.79	74.90	-46.26	-96.94	80.59
Upupa_epops	**90**	90.86	119.91	-49.96	407.44	88.93	-59.18	-96.13	4.98	96.24	-88.35	-99.97	-41.64	76.00	-17.04	-55.71	22.89	77.48	-40.10	-90.82	37.79
Vanellus_indicus	49	87.60	27.71	-13.98	74.09	100.00	337.35	162.61	539.82	100.00	116.71	52.54	179.44	89.71	-15.31	-33.89	3.64	99.98	25.68	12.33	39.23
Vanellus_malabaricus	59	75.11	41.65	-60.48	173.51	91.85	-70.70	-97.62	-8.54	96.69	-94.37	-99.99	-61.84	61.63	-7.91	-51.08	39.38	86.69	-56.32	-95.70	18.92
Zosterops_palpebrosus	**28**	53.46	-5.98	-83.21	137.38	69.00	-43.22	-98.48	133.97	96.49	-95.57	-100.00	-58.46	84.74	-30.38	-71.83	14.52	94.50	-77.14	-99.23	-24.89

Supplementary Table 9. Effects of the transition to ZBNF, and of farming system-wise effects of increasing landscape-level agricultural productivity and of economic profit for each trophic guild. The posterior distribution (PD) and estimated percentage change in abundance (with lower and upper 95% Bayesian credible intervals) are shown.

	**Effect of the transition to ZBNF**				**Effect of agricultural productivity in ZBNF systems**				**Effect of agricultural productivity in agrichemical systems**				**Effect of economic profit in ZBNF systems**				**Effect of economic profit in agrichemical systems**			
**Trophic guild**	**PD**	**% change**	**% change lower CI**	**% change upper CI**	**PD**	**% change**	**% change lower CI**	**% change upper CI**	**PD**	**% change**	**% change lower CI**	**% change upper CI**	**PD**	**% change**	**% change lower CI**	**% change upper CI**	**PD**	**% change**	**% change lower CI**	**% change upper CI**
Frugivore	99.21	160.25	9.47	374.10	78.50	-33.68	-82.18	29.93	100.00	-97.72	-99.97	-91.74	81.61	29.21	-24.72	89.99	100.00	-93.10	-99.09	-81.98
Granivore	77.55	-20.25	-55.80	21.97	86.19	-40.28	-79.34	13.21	85.01	-48.48	-90.34	17.77	99.05	-38.99	-60.20	-15.57	97.43	-51.76	-77.76	-19.21

Invertivore	97.68	48.98	1.44	100.69	81.13	-24.60	-63.33	18.92	99.99	-85.89	-96.36	-70.61	92.86	-24.17	-48.28	1.20	99.95	-56.86	-77.71	-36.45
Omnivore	50.39	-0.20	-33.28	33.91	75.55	-18.29	-56.47	23.74	100.00	-82.23	-94.30	-67.27	88.11	-15.65	-35.16	5.99	99.99	-56.56	-74.36	-36.46
Vertivore	98.19	80.85	0.89	176.85	99.88	189.25	38.95	393.35	94.83	-58.09	-87.86	-13.40	93.33	-21.89	-44.15	-0.28	99.16	-52.23	-76.16	-25.05

Supplementary Table 10. Complete set of interview questions.

1. **Background and socioeconomic details.**
    a. What is your name?
    b. What is the name of your village?
    c. How many dependents are there in your household (i.e., people eating from the same cooking pot)? Please state the number of adults and children

2. **Fields managed and crops grown**
    a. How many fields do you own or manage?
    b. What is the size of each field?
    c. Which crops did you grow in each field in each season in 2022?
        i. Were the crops grown representative of what you grew in the previous four calendar years, or did you grow different crops?
            1. If the latter, please state what the differences were?
    d. Which of these is the field that falls within our study site ('focal field')?
    e. For the field in our study site (focal field), which crops did you grow in each season over the last five years (or as long as you can remember)?
    If you can, please include the variety.
        i. Was it the main crop or an intercrop?
        i. Was the crop is grown in the whole field or only a fraction of it?
            1. If the latter, how big was the area of where the crop was grown?
        If unknown, ask for the total number of plants planted and the number of plants in 1 m^2
        ii. Was the crop seeded/planted in the same growing season it was harvested in?
            1. If not, please state the month and year the crop was sown/planted.
            2. If not, please state the age of the crop when you started harvesting it.

iii. For each crop in each season, how many kilograms did you harvest?
   If you can, please include both the weight at harvest and the weight after processing the crop (e.g., after drying, dehusking, deshelling, i.e. as sold on the market).
   1. What proportion (or how many kilogrammes) did you keep for your own consumption and what proportion did you sell at the market?
      Please state if this is the proportion of the weight at time of harvest or sold at the market.
   2. For how much (INR) did you sell the crop at the market?
      Please include the unit of measurement, e.g., per bag, per kg, total harvest
   3. What is the current price per kilogramme?
   4. Did you receive a price premium?
      a. If so, please state why.

iv. Did you grow any non-food plants? E.g, marigold, fodder grasses, native trees or bushes
   1. If so, which and how many?
      Please include the unit of measurement; e.g. number of plants, area of coverage
   2. Why did you grow these plants? *E.g. medicinal, fodder, habitat for natural enemies/pollinators*
   3. Where did you get the seeds/seedlings from?
      a. If you bought them, how much did you pay?
         Please include the unit.
   4. Did you sell the plants or its products?
      a. If so, for how much?

b. Are there any trees, bushes, or other vegetation in the approximately 1 km surrounding any of your fields from which you collect any items or make use of in any other way? *For example, coconuts, neem, tamarind, toddy, palm wine, grasses for fodder, firewood, grazing for livestock etc.*
   i. If so, please specify the plant, the item and quantity collected, the intended use, and whether the plants were planted (by you or other villagers).

3. **Ownership.** Questions concern the focal field only.
   a. Are you the owner of the field or do you have to pay any rent, lease, tax, or similar for the field?
   b. How much are you paying (in rupees and/or fraction of crop harvested)? Please include the time period.

4. **Seeds**. Questions concern the focal field only. Please answer the following questions for each growing season the past five calendar years. Please specify the unit used.
   a. Where did you get the seeds/seedlings from?
   b. How many seeds or seedlings did you plant?

c. Did you buy them?
    i. If so, how much did you pay (per unit or in total)?

5. **Irrigation**. Questions concern the focal field only. Please answer the following questions for each calendar year or for each 'round' per year for the past five calendar years. Please specify the unit used.
    a. Do you irrigate your field?
        i. If so, how much water do you use (please state the unit)?
        ii. If so, how much do you pay for the water

6. **Inputs.** Questions concern the focal field only. Please answer the following questions for each calendar year or for each 'round' per year for the past five calendar years. Please specify the unit used.
    a. Did you use any form fertilizer or other form of soil treatment?
        i. If so, what is the name or type?
        ii. Did you buy or make it yourself?
        iii. What ingredients does it contain?
        iv. How many kg or litres did you apply per round?
        v. How many rounds did you conduct in the year?
        vi. What is the cost per kg/litre/bottle/round?
        If per bottle, please state how many litres/kg there are in a bottle.
        If hand-made but you had to purchase the ingredients, how much did you pay for the ingredients?
    b. What other substances did you add to your field?
    This may include herbicides ("weedicides"), fungicides, and insecticides, including any other hand-made or locally sourced solutions.
        i. Did you buy or make it yourself?
        ii. What ingredients does it contain?
        iii. Why did you apply it? *E.g. to control pests*
        iv. How many kg or litres did you apply per round?
        v. How many rounds did you do in the year?
        vi. What is the cost per kg/litre/bottle?
        If per bottle, please state how many litres/kg there are in a bottle.
        If hand-made but you had to purchase the ingredients, how much did you pay for the ingredients?

7. **Farming practice.** Questions concern the focal field only.
Questions on input, intercropping, and costs and are covered separately.

Please provide information for 2022. If what you did in 2022 differs from what you did in the previous four years differs then please provide information for these years separately.

a. Since when have you farmed the field?
b. Do you have a name for the farming practice you are using?
    i. If so, what is it?
    ii. If ZBNF, can you please state and briefly describe the 'wheels' of ZBNF you are using? You may refer to the inputs you described above.
c. Did you use cover crops?
    1. If so, which ones?
d. Did you till or plough your field?
    i. If so, how often?
e. Did you install any bird perches?
f. Did you install any traps to catch pests?
g. Did you do anything else to attract pollinators and/or to prevent or limit damage by pests and/or disease?
h. Have you always farmed with the methods you used in 2022?
    i. If not, when did you change the management? *E.g. transition to ZBNF*
    ii. If not, how did you manage the field before?
    Please give a very brief description, stating whether you added any inputs, what the ingredients were, whether they represent agro-chemicals, if you used cover and/or intercrops, and if you tilled the land.
i. Do you know how the field was managed before you did (by either a family member or previous tenant)?
    i. If so, please give the same details as above (3.h.ii) and include the time period (YY – YY).
j. Do you know when the land was cleared for agriculture?
    i. If so, please state when.
k. Are you managing your other fields using the same farming practice?

8. **Equipment.** Please answer the following questions for each calendar for the past five years.
Since one set up equipment is likely to be used for all fields, please provide the information for all your fields (rather than the focal field only).
    a. What farm equipment did you buy? For example, machete, axe, digging hoe, sharpening stone, bucket, irrigation pipes, sacks, boots, socks
    b. How many pieces of each equipment did you buy?
    c. How much did you pay for each item?
    Please state also state the current price if it differs from what you paid.
    d. How long do you expect each item to last before you have to buy a new one?

e. Is there any other equipment that you use but haven't bought in the last five years? E.g. tractor, sprayer
    i. If so, please provide the same details as above.
f. Did you rent any equipment or livestock?
    i. If so, what item(s) or livestock did you rent and how many of each?
    ii. How much did you pay per rental?
        1. If the equipment requires petrol or electricity to run, please state whether this is included in the rental price.
    iii. How many times did you rent it per year?

9. **Livestock.**
    a. Do you own any livestock and/or poultry?
        i. If so, which ones and how many of each category? How much did you pay for their feed every year for the past five years?
        ii. How much did you pay for their veterinary bills every year for the past five years?
        iii. How much did you pay for their shelter every year for the past five years?
        iv. Did you pay anything else for their upkeep?
            1. If so, please specify the type and amount (in INR).
        v. How much would you be able to sell them for?
        vi. How long do they live?
        vii. Did you use them to manage your fields? *E.g. cows to plough the land*
            1. If so, how many times did you use them each year?
            You can break your answer down by season if needed.
            2. Each time you used them, how many days did you use them for?
            3. How many animals did you use?
        viii. Do you use any of their products (e.g., cow dung or urine) to grow your crops?
            1. If so, are theses the ones discussed in Q6?
                a. If not, please specify.

10. **Labour.** Questions concern the focal field only. Please answer the following questions for each calendar year for the past five years. If unable to answer per year, then specify if the information is per growing season or per month.
    a. What is the local basic daily wage rate?
    b. Please indicate if you conducted the following tasks at any point:
        i. Clearance of leaf litter and twigs
        ii. Tree felling
        iii. Pruning
        iv. Burning

v. Tilling
vi. Mulching
vii. Irrigating
viii. Spraying
ix. Fertilising
x. Weeding
xi. Carrying and planting/sowing
xii. Levelling (the ground)
xiii. Harvesting
xiv. Processing of crop
xv. Transporting to point of sale
xvi. Selling

c. How many people were involved (including you if you took part)?
d. Were the other people paid or unpaid?
e. How many days did it take per round?
f. How many rounds were there per year?
g. Were there any other tasks that we haven't discussed yet?
    i. If so, please provide the same details as for the other tasks.

11. **Energy.** Questions concern the focal field only. Please answer the following questions for each calendar year for the past five years. If unable to answer per year, then specify if the information is per growing season or per month.
    a. Do you use any electricity, petrol, or gas to perform any of the tasks described above?
        i. If so, how much did you use? Please include the unit.
        ii. How much did you pay (per unit)?

12. **Transport.** Please answer the following questions for each calendar year for the past five years. If unable to answer per year, then specify if the information is per growing season or per month. Please state if the information is for the crops grown at the focal field only, or for crops from all your fields.
    a. What is the cost of transportation to the point of sale (other than labour)? *E.g. fuel cost.*
        i. *Please specify how many units (e.g. bags, kg) that covers.*
