# Supplementary material for: India’s agroecology programme, ‘Zero Budget Natural Farming’, delivers biodiversity and economic benefits without lowering yields
Source: Nat Ecol Evol. 2025 Sep 19;9(11):2057–68. doi: 10.1038/s41559-025-02849-7 (PMC12592218; doi:10.1038/s41559-025-02849-7)
Supplement: Supplementary file 2 — Reporting Summary [file 41559_2025_2849_MOESM2_ESM.pdf]

nature portfolio

Corresponding author(s): Berger, Iris

Last updated by author(s): 2025/08/02

Reporting Summary

Nature Portfolio wishes to improve the reproducibility of the work that we publish. This form provides structure for consistency and transparency in reporting. For further information on Nature Portfolio policies, see our Editorial Policies and the Editorial Policy Checklist.

Statistics

For all statistical analyses, confirm that the following items are present in the figure legend, table legend, main text, or Methods section.

n/a	Confirmed	
☐	☒	The exact sample size (*n*) for each experimental group/condition, given as a discrete number and unit of measurement
☐	☒	A statement on whether measurements were taken from distinct samples or whether the same sample was measured repeatedly
☐	☒	The statistical test(s) used AND whether they are one- or two-sided<br>*Only common tests should be described solely by name; describe more complex techniques in the Methods section.*
☐	☒	A description of all covariates tested
☐	☒	A description of any assumptions or corrections, such as tests of normality and adjustment for multiple comparisons
☐	☒	A full description of the statistical parameters including central tendency (e.g. means) or other basic estimates (e.g. regression coefficient) AND variation (e.g. standard deviation) or associated estimates of uncertainty (e.g. confidence intervals)
☐	☒	For null hypothesis testing, the test statistic (e.g. *F*, *t*, *r*) with confidence intervals, effect sizes, degrees of freedom and *P* value noted<br>*Give P values as exact values whenever suitable.*
☐	☒	For Bayesian analysis, information on the choice of priors and Markov chain Monte Carlo settings
☐	☒	For hierarchical and complex designs, identification of the appropriate level for tests and full reporting of outcomes
☐	☒	Estimates of effect sizes (e.g. Cohen's *d*, Pearson's *r*), indicating how they were calculated

Our web collection on statistics for biologists contains articles on many of the points above.

Software and code

Policy information about availability of computer code

Data collection	QGIS version 3.38.3 and Google Earth Engine version 7.3 were used to aid in the selection of field sites. The outcome, primary predictor, and some covariate data were obtained through field studies. Area measurements of different land-uses were conducted in QGIS version 3.38.3 (see Supplementary Information 3). The other covariate data were downloaded from various sources online (see Supplementary Information 2 and Supplementary Table 2) and processed in Google Earth Engine version 7.3.All data were collated and cleaned in R version 4.3.1.
Data analysis	All analyses were carried out in R version 4.3.1. The key packages used were: "ape", "boot", "brms", "car", "DHARMa", "dplyr", "ggplot2", "lme4", "MatchIt", "mrds", "tidybayes", and "vegan".Aesthetic figure edits were conducted using Inkscape version 1.4.<br>The code is available on GitHub: https://github.com/irisberger/ZBNF

For manuscripts utilizing custom algorithms or software that are central to the research but not yet described in published literature, software must be made available to editors and reviewers. We strongly encourage code deposition in a community repository (e.g. GitHub). See the Nature Portfolio guidelines for submitting code & software for further information.

Data

Policy information about availability of data

All manuscripts must include a data availability statement. This statement should provide the following information, where applicable:

- Accession codes, unique identifiers, or web links for publicly available datasets
- A description of any restrictions on data availability
- For clinical datasets or third party data, please ensure that the statement adheres to our policy

The interview and bird data used in this study are available via Zenodo at: 10.5281/zenodo.16687021

Research involving human participants, their data, or biological material

Policy information about studies with human participants or human data. See also policy information about sex, gender (identity/presentation), and sexual orientation and race, ethnicity and racism.

Reporting on sex and gender	Data on sex and gender were not collected.
Reporting on race, ethnicity, or other socially relevant groupings	For our main analyses, we sampled from 'plain' and 'tribal' areas equally. Traditional and indigenous farming practices, low accessibility, and a high proportion of subsistence farmers characterise "tribal" areas. In contrast, 'plain' areas, which are nearer to the coast and better connected to agricultural markets, are dominated by farming systems that have adopted high use of pesticides, fertilisers, irrigation, and agricultural credit. However, as a robustness check, we also conducted separate analyses for 'tribal' and "plain" areas (see Supplementary Information 6).
Population characteristics	The participants were smallholder farmers that typically kept a proportion of their harvest for their own consumption and sold the remainder.
Recruitment	At each study landscape (square), we interviewed the farmers managing the fields upon which our four equally spaced points fell, as well as up to two more randomly selected farmers per landscape (see Supplementary Information 3). No farmers refused to be interviewed. We accounted for observable confounders by careful selection of study landscapes and statistical matching (see Methods and Supplementary Information 2).
Ethics oversight	Ethical approval was given by the Cambridge Psychology Research Ethics Committee (approval code PRE.2022.090) at the University of Cambridge prior to commencement. Before participating in the study, all farmers gave informed consent.

Note that full information on the approval of the study protocol must also be provided in the manuscript.

Field-specific reporting

Please select the one below that is the best fit for your research. If you are not sure, read the appropriate sections before making your selection.

☐ Life sciences ☐ Behavioural & social sciences ☒ Ecological, evolutionary & environmental sciences

For a reference copy of the document with all sections, see nature.com/documents/nr-reporting-summary-flat.pdf

Ecological, evolutionary & environmental sciences study design

All studies must disclose on these points even when the disclosure is negative.

Study description	We collected yield and farm management data from ‘zero budget natural farming’ (ZBNF) and agrichemical farmers, and conducted bird counts in these two systems as well as natural forests. We assessed the impact of ZBNF on yield, profit, and densities of different bird species, and we evaluated the associations between landscape-level yield, profit, and bird population outcomes for each farming system.
Research sample	In total, we worked in 51 landscapes (13 ZBNF landscapes, 13 agrichemical landscapes, 25 forest landscapes). We collected yield data from 206 harvests and profit data from 128 fields found within those landscapes, and we obtained density estimates of 199 bird species by conducting 816 point counts. We recorded all bird species present at our sites. Our samples are representative of ZBNF and agrichemical farming systems, and of natural forests in northern Andhra Pradesh.
Sampling strategy	We required large continuous areas to be exclusively farmed using ZBNF practices, and further site requirements to reduce the influence of possible confounders left us with 13 landscapes. We identified agrichemical and forest sites with similar biophysical and socioeconomic characteristics (see Supplementary Information 2 for details).
Data collection	We conducted 10-minute point counts and identified all birds present from visual and/or acoustic cues. We collected yield and profit data via a questionnaire with the farmers. See methods for details.
Timing and spatial scale	We conducted 128 structured interviews of farmers between December 2022 and February 2023. The interviews asked for detailed information about each identified field over a one-year recall period (beyond that recall issues may arise, see Supplementary Information 3). We conducted 10-minute point counts with no settling-in period at each point location in both the winter

	(December–March) and summer (April–June) seasons over two years (2021/2022 and 2022/2023). Hence, we visited each point on four separate occasions, totalling 816 repeats (51 squares * 4 points * 4 repeats) and 8,320 minutes observation (160 minutes per square) across all sites.
Data exclusions	Individual birds we only saw in flight and birds that flew in during the count period were excluded, and we removed observations greater than 100 metres from the point. We discarded records of bird species that point counts do not adequately sample, namely largely aerial and/or transient species (see Supplementary Information 4). We removed four tapioca harvests when examining ZBNF's impact on yield as they represented outliers. For the profit analysis, we removed seven fields that had estimated profits of over 25,000 INR per hectare as these were deemed implausible.
Reproducibility	No experiments were conducted.
Randomization	Farmers chose whether or not to adopt ZBNF prior to our study. We used statistical matching to control for observable confounders (see Methods).
Blinding	Farmers chose whether or not to adopt ZBNF prior to our study.

Did the study involve field work? ☒ Yes ☐ No

Field work, collection and transport

Field conditions	Surveying birds in both the winter and summer seasons over two years allowed us to record species mainly vocal during the breeding season as well as winter migrants, and to control for seasonal and yearly variability. We avoided conditions of rain, fog, or high winds.
Location	Andhra Pradesh, India (districts: East Godavari, West Godavari, Vizianagaram, Visakhapatnam, and Srikakulam)
Access & import/export	Research was approved by ethics committees at the University of Cambridge (approval code PRE.2022.090). We did not work in strictly protected areas (IUCN category II).
Disturbance	Disturbance to birds and other taxa was minimised by using survey methods that are not invasive (i.e. purely observational).

Reporting for specific materials, systems and methods

We require information from authors about some types of materials, experimental systems and methods used in many studies. Here, indicate whether each material, system or method listed is relevant to your study. If you are not sure if a list item applies to your research, read the appropriate section before selecting a response.

Materials & experimental systems

n/a	Involved in the study	
☒	☐	Antibodies
☒	☐	Eukaryotic cell lines
☒	☐	Palaeontology and archaeology
☐	☒	Animals and other organisms
☒	☐	Clinical data
☒	☐	Dual use research of concern
☒	☐	Plants

Methods

n/a	Involved in the study	
☒	☐	ChIP-seq
☒	☐	Flow cytometry
☒	☐	MRI-based neuroimaging

Animals and other research organisms

Policy information about studies involving animals; ARRIVE guidelines recommended for reporting animal research, and Sex and Gender in Research

Laboratory animals	The study did not involve laboratory animals.
Wild animals	199 bird species were observed during point counts. See Supplementary Table 6 for a full species list.
Reporting on sex	No sex-based analyses were performed.
Field-collected samples	The study did not involve samples collected from the field.
Ethics oversight	No ethical approval was required for the bird surveys as these were entirely observational.

Note that full information on the approval of the study protocol must also be provided in the manuscript.

Plants

Seed stocks	No plant material was collected.
Novel plant genotypes	Not applicable.
Authentication	Not applicable.
